# Temporal Effects of Disease Signature Genes and Core Mechanisms in the Hyperacute Phase of Acute Ischemic Stroke: A Bioinformatics Analysis and Experimental Validation

**DOI:** 10.1155/mi/6808184

**Published:** 2025-06-06

**Authors:** Peng-Li Ding, Kai-Xin Zhang, Fang Yao, Wen-Qiang Cui, Zhen-Ling Liu, Yi-Ran Wang, Xiang-Ying Wang, Wei Liu, Heng-Ye Zhao, Hong-Yun Wu, Ya-Han Wang, Xiang-Qing Xu

**Affiliations:** ^1^First College of Clinical Medicine, Shandong University of Traditional Chinese Medicine, Jinan, Shandong, China; ^2^Nursing Department, Shandong University of Traditional Chinese Medicine Affiliated Hospital, Jinan, Shandong, China; ^3^Neurology Department, Shandong University of Traditional Chinese Medicine Affiliated Hospital, Jinan, Shandong, China; ^4^Department of Neurology, Hunan Provincial Hospital of Integrative Medicine, Changsha, Hunan Province, China

**Keywords:** acute ischemic stroke, apoptotic dynamics, bioinformatics analysis, hyperacute phase, temporal regulation, toll-like receptor signaling

## Abstract

**Background:** The pathophysiological progression during the hyperacute phase of acute ischemic stroke (AIS) critically determines clinical outcomes. Identification of phase-specific biomarkers and elucidation of their temporal regulatory mechanisms are pivotal for optimizing therapeutic interventions.

**Methods:** Disease signature genes and their mechanisms of action were screened based on the Gene Expression Omnibus database. This involved the use of differentially expressed gene screening, weighted gene co-expression network analysis, Mfuzz analysis, Gene Ontology, Kyoto Encyclopedia of Genes and Genomes enrichment analysis, support vector machines, random forest algorithms, and gene set enrichment analysis. The expression of disease-characteristic genes and their related mechanisms were further validated in both in vivo and in vitro models.

**Results:** Six hyperacute-phase signature genes (*Pip5k1c*, *Nlgn2*, *Fzd2*, *Cd86*, *Agpat1*, and *Degs2*) were identified in the hyperacute phase of AIS. In light of the gene effect mechanism, the regulation of the neuroinflammatory response and apoptosis by the TLR2/TLR4/NF-*κ*B pathway was monitored in the hyperacute phase of AIS at three times: 3, 6, and 12 h. The results indicated a progressively intensified neuroinflammatory response and the fluctuating growth of early apoptosis changes.

**Conclusion:** This study systematically identifies hyperacute-phase-specific biomarkers in AIS and delineates their temporal regulatory logic. The time-course dynamics of neuronal apoptosis and inflammatory regulation in the hyperacute phase of AIS were monitored. The observed biphasic apoptotic pattern provides mechanistic insights for developing chronologically targeted therapies, such as timed inhibition of TLR4/CD86 during 0–3 h to block inflammatory initiation, or administration of Agpat1 agonists at 3–6 h to stabilize mitochondrial function. These findings help alleviate the current ‘molecular blind spot' in early stroke diagnosis and intervention.

## 1. Background

As the second leading cause of death and the third leading cause of disability worldwide, acute ischemic stroke (AIS) is the most common cause of death [1]. AIS occurs when there is an inadequate blood supply to large cerebral arteries or their branches. This inadequacy leads to a lack of oxygen and energy, triggering a series of ischemic cascade reactions [2, 3], ultimately leading to the formation of a core infarct core and ischemic penumbra. “Time is brain” has always been an important guideline in the clinical treatment of AIS. After cerebral infarction, the brain loses 1.9 million neuronal cells/min, and the number of neuronal cells lost per 1 h delay is equivalent to 3.6 years of normal aging [4]. Therefore, a reduction in the onset-to-treatment time of stroke is strongly associated with a favorable prognostic outcome [5]. For this reason, the hyperacute phase of AIS is considered to occur within 6 h of onset, and some imaging studies include cases within 12 h [6], with timely diagnosis and treatment in the hyperacute phase being closely related to the clinical outcome of patients with AIS.

Biomarker and genomic analyses of AIS have been studied extensively [7, 8] and provide important diagnostic basis and therapeutic targets. Recent advances highlight dynamic biomarkers such as mitochondrial membrane potential (MMP)-9, which predicts hemorrhagic transformation risk during endovascular therapy, and neutrophil-derived metabolites (e.g., 13,14-dihydroretinol) linked to ischemic tolerance via FoxO signaling [9]. Additionally, noninvasive cardiac-specific biomarkers (e.g., hs-TnI) have shown promise in predicting vascular stenosis and stroke outcomes [10]. However, while inflammation (NLRP3 inflammasome activation) [11] and apoptosis [12] are well-characterized in subacute/chronic phases, molecular signatures specific to the hyperacute phase (<6 h) remain underexplored, particularly those capturing early neuroinflammatory priming and neuronal apoptosis initiation. The lack of reliable biomarkers capturing initial pathogenic events (e.g., neuroinflammatory priming and neuronal apoptosis initiation) during this critical time window hinders the advancement of precision medicine in AIS. Therefore, systematic screening of temporally-specific disease-characteristic genes and dynamic evaluation of early pathological changes are urgently needed.

In this study, the core disease genes and their mechanisms of action in the hyperacute phase were explored by bioinformatics analysis, and the changes in the expression of the disease signature genes and the effect of pathogenesis at multiple time points in the hyperacute phase were further verified by in vivo and in vitro experiments to provide a theoretical foundation for the diagnosis and treatment of the hyperacute phase of AIS.

## 2. Methods

### 2.1. Biological Information Analysis

#### 2.1.1. Data Sources

“Cerebral infarction” was used as the keywords to retrieve the gene expression profile retrieved from the GEO database (http://www.ncbi.nlm.nih.gov/geo), the GSE212732 dataset (GPL17117) was obtained, including four cases in the control group, three cases in MCAO_3h group, four cases in MCAO_6h group and four cases in MCAO_12h group (*Rattus norvegicus*).

#### 2.1.2. Chip Data Processing and Differentially Expressed Genes (DEGs) Screening

To visualize the chip data after quality control, the “ggplot2” package was used. The R language “limma conductor” software package analyzed chip data (|log2FC| > 1, Adjust_*p*-value < 0.05). The volcano map of the DEGs was drawn using “ggplot 2” package.

#### 2.1.3. Gene Set Enrichment Analysis (GSEA)

The gmt dataset accessible via MsigDB database (https://www.gsea-msigdb.org/gsea/index.jsp) was utilized as the reference gene set. The default weighted enrichment statistical method was used to analyze the potential effect pathways in the acute period of cerebral infarction, and “ggplot2” was used to visualize the top five results of enrichment. The absolute number of normalized enrichment scores (NES) > 1, false discovery rate (FDR) < 0.25, and Adjust_*p*-value < 0.05 were used as the selection criteria.

#### 2.1.4. Weighted Gene Co-expression Network Analysis (WGCNA)

The co-expression network was constructed for all genes in the dataset using the WGCNA-R package, the genes in the top 60% of the variance were filtered, and the soft threshold was established at a value of 8. The weighted adjacency matrix was transformed into a topological overlap matrix (TOM) for the purpose of estimating network connectivity, and the hierarchical clustering method was then employed for the construction of the cluster tree structure of the TOM.

#### 2.1.5. Mfuzz Analysis

The fuzzy C-means clustering (FCM) algorithm was used to analyze the time trend of gene expression (Mfuzz: a software package designed for the soft clustering of microarray data). Sequencing data at different time points in the GSE212732 dataset were analyzed for gene timing expression patterns through the http://www.bioinformatics.com.cn/ website.

#### 2.1.6. Identification of Significant Disease-Related Genes

Gene comparison analysis was performed based on the results of WGCNA, DEGs, and Mfuzz analysis to obtain the intersection genes of the three, and the final intersection genes were determined using a review in the Uniprot database (Swiss-Prot).

#### 2.1.7. Gene Ontology (GO) and Kyoto Encyclopedia of Genes and Genomes (KEGG)

ClusterProfiler was employed for functional annotation of the disease-characteristic genes. GO and KEGG were employed to evaluate the pertinent functional classes, and enrichment pathways (*p* < 0.05) were considered as significant classes.

#### 2.1.8. Support Vector Machines (SVM) and Random Forest (RF) Algorithm

An algorithm (recursive feature elimination) was used to select the best genes from the metadata queue. In this study, the SVM algorithm was completed through the “e1071,” “kernlab,” and “caret” packages in R. The RF algorithm was completed using the “random forest” package in R, and the core genes screened from SVM and RF were intersected by the Venn package and defined as disease-characteristic genes.

#### 2.1.9. GSEA of Characteristic Genes

Based on the median gene expression, the samples in the disease group chip data were dichotomized into two groups with high and low expression, respectively. Subsequently, the single-gene GSEA was performed. Absolute values of NES > 1 and Adjust_*p*-value < 0.05 were used as the selection criteria.

### 2.2. In Vivo Animal Experiments and In Vitro Cell Culture

#### 2.2.1. Permanent Middle Cerebral Artery Occlusion (pMCAO) Model Preparation

Sixty specific pathogen-free (SPF) male Sprague-Dawley rats (8-week-old, 250 ± 20 g, purchased from Beijing Viton Lihua Laboratory Animal Technology Company (License No.: SCXK (Zhe)2021-0006)) were stratified by body weight into four groups (*n* = 15/group) using a random number table: Sham, 3, 6, and 12 h pMCAO groups. All rats were acclimatized for 7 days pre-surgery under controlled conditions (12 h light/dark cycle, 22°C ± 1°C, 55% ± 5% humidity) with ad libitum access to sterilized food and water.

The pMCAO model was performed using the wire embolization method. Aphrodite (ready-to-use tribromoethanol) (Meilunbio, MA0478) was used for inhalation anesthesia. For proper occlusion of the middle cerebral artery (MCA), the internal carotid artery (ICA), external carotid artery (ECA), and right common carotid artery (CCA) were meticulously separated, and then the CCA was dissected. Next, an 18–20 mm long, 0.285 mm diameter polylysine-coated monofilament nylon suture was passed through the ICA. Insertion of the nylon suture was made for 18.5 ± 0.5 mm to the point of a slight sense of resistance for the nylon suture to be passed through the ICA at the head. The inserted 18.5 ± 0.5 mm suture provided a slight sense of resistance such that the head of the nylon suture passed through the beginning of the MCA and reached the thinner anterior cerebral artery, which completes the occlusion of the right MCA. The ICA was ligated to secure the nylon suture and prevent hemorrhage, and the sutures were placed layer-by-layer. Body temperature was maintained at 37.0°C ± 0.5°C using a feedback-controlled heating pad throughout surgery. The rats in the 3, 6, and 12 h groups were assessed using modified Neurological Severity Score (mNSS ≥ 6 included), and brain tissues were removed 3, 6, and 12 h after the pMCAO operation.

#### 2.2.2. MCAO Model Establishment and Evaluation

A mNSS was administered to all rats according to the established groups. mNSS is a comprehensive method for assessing neurological function based on motor, sensory, balance, and reflex measurements, and the score ranged from 0 to 18, with a normal score of 0 and a maximal deficit score of 18. The higher scores indicated greater nerve damage [13, 14].

#### 2.2.3. 2,3,5-Triphenyl Tetrazolium Chloride (TTC) Staining

Following anesthesia of the rats, the brains were peeled off on ice, and rinsed with saline, and then the brain tissues were placed in a refrigerator at −30°C for 15 min after rapid freezing and sliced. The brain slices were placed in 2% TTC staining solution, protected from light, and incubated at a constant temperature of 37°C for 20 min, during which the brain slices were turned over for 1–2 times and then washed with ddH_2_O. After staining, the normal brain tissue exhibited a red coloration, while the infarcted area displayed a white hue. Images were collected using a digital camera, and the percentages of infarct volume and brain edema volume were measured using ImageJ software. Percentage of infarct volume = infarct volume/whole brain volume × 100%.

#### 2.2.4. Histological and Nissl Staining

Following the administration of anesthesia, rats were subjected to intracardiac perfusion of saline until the liver turned white, and then the brains were peeled off, fixed with 4% paraformaldehyde for 24 h, dehydrated, wax-immersed, embedded, and sliced into 5 μm slices. The sections were deparaffinized and subsequently stained with the hematoxylin and eosin staining (H&E staining) [15] and Nissl staining [16]. The stained sections were observed under a fluorescence microscope, and images were captured. Subsequently, the relevant parts of the samples were analyzed using the ImageJ software.

#### 2.2.5. Terminal Deoxynucleotidyl Transferase-Mediated dUTP Nick End Labeling (TUNEL) Staining

Sections of rat brain were taken according to a previous description and incubated with a methanol solution containing 0.2% H_2_O_2_ for 0.5 h to block endogenous peroxidase activity, followed by TUNEL staining (PF00009, Proteintech) according to the manufacturer's instructions. Images were acquired by fluorescence microscopy and quantified using ImageJ software with a tuning index of (TUNEL-positive cells)/(total number of cells) × 100%.

#### 2.2.6. Enzyme-Linked Immunosorbent Assay (ELISA)

ELISA kits were used to detect the Toll downstream-associated inflammatory markers interleukin-1*β* (IL-1*β*) (Beyotime, RatIL-1*β* ELISA Kit, PI303), interleukin-6 (IL-6) (Beyotime, RatIL-6 ELISA Kit, PI328), and tumor necrosis factor-*α* (TNF-*α*) (Beyotime, RatTNF-*α* ELISA Kit, PT516) expression levels were quantified according to the manufacturer's specifications. The procedure included the dissection of brain tissue, lysis, the extraction of supernatants, and ELISA assays to quantify the expression levels of IL-1*β*, IL-6, and TNF-*α* in Sham and 12 h groups.

#### 2.2.7. Immunofluorescence (IF)

Brain sections of from the rats in the Sham and 12 h groups were subjected to antigen repairand blocked with bovine serum albumin (BSA). Subsequently, primary antibodies TNF-*α* (Proteintech, 60291–1-Ig), IL-6 (Proteintech, 23457–1-AP), and IL-1*β* (Abcam, ab315084) were applied. Subsequently, the goat anti-rabbit secondary antibody was incubated at 4°C for 1 h. Nuclei were counterstained with DAPI. The sections were visualized by fluorescence microscopy.

#### 2.2.8. Cells and the Establishment of the Oxygen-Glucose Deprivation (OGD) Model

The high differentiation rat adrenal pheochromocytoma cell line, PC-12 (Typical Cultures Preservation Committee of the Chinese Academy of Sciences Cell Bank, SCSP-517), was utilized. And PC-12 cells-specialized medium (RPMI 1640 medium supplemented with 10% fetal bovine serum and 1% penicillin/streptomycin) were purchased from Procell Life Science & Technology Co., Ltd (Wuhan, China). The cells were divided into four experimental groups, designated as control, 3, 6, and 12 h groups. The culture medium of the experimental group was replaced with a sugar-free medium, and the cells were anoxically treated in a triple-air incubator with 95% high-purity nitrogen (N_2_) for 3, 6, and 12 h, respectively, whereas the cells in the control group were maintained in the same environment without any change.

#### 2.2.9. Western Blotting

Rat brain tissue and cells were collected separately. Total protein was extracted using a radioimmunoprecipitation assay (RIPA). A bicinchoninic acid (BCA) protein assay kit was used to determine the protein concentration, and the protein samples were subjected to sodium dodecyl sulfate-polyacrylamide gel electrophoresis (SDS-PAGE), transferred to nitrocellulose membranes, closed with 5% skimmed milk for 2 h, and then incubated with the primary antibody overnight. Cleaved-Caspase3 (cl-Caspase3) (1 : 1000; #9964; CST), Bax (1 : 5000; ab32503; Abcam), Bcl2 (1 : 1000; #3498; CST), TLR2 (1 : 1000; ab209217; Abcam), TLR4 (1 : 1000; 3G9A4; Proteintech), NF-kB p65 (Phospho) (1 : 1000; ab76302; Abcam), NF-kB p65 (1 : 1000; #8242; CST), and *β*-actin (1 : 10,000, HRP-60008, Proteintech), and the membranes were incubated for 1 h in secondary antibodies (1 : 5000, RGAR001, Proteintech), (1 : 5000SA00001-1, Proteintech), and the gray values of the blotted protein bands were analyzed using the ImageJ software.

#### 2.2.10. Real-Time Quantitative Polymerase Chain Reaction (RT-PCR)

Total RNA was extracted from distinct groups of rat brain tissues and PC-12 cells in accordance with the instructions provided by the manufacturer (SPARKJADE, AG304, AG0501, AG0402, China). First-strand cDNA synthesis was conducted in order to obtain the cDNA products. Polymerase chain reaction (PCR) solutions were prepared using appropriate amounts of forward and reverse primer, 2×SYBRqPCRmix, RNase-freeH_2_O, and cDNA. The two-step PCR cycles were as follows: hold phase: 94°C for 2–3 min; PCR phase: first step: 94°C for 10–20 s; second step: 60°C for 34 s, for a total of 40 cycles. The results were subjected to analysis using the relative quantitative 2-^ΔΔ^CT method. Correlation sequences are designed as primers for real-time PCR (RT-PCR) (Table 1).

#### 2.2.11. Cell Counting Kit-8 (CCK-8)

The viability of the cells was determined through the use of a cell CCK-8 assay (Meilunbio, Dalian, China). PC-12 cells were seeded in 96-well plates at a density of ~1 × 104 cells/well and cultured in a cell culture incubator for 24 h. After good growth, the cells were incubated in glucose-free DMEM at 37°C, 5% CO_2_, and 95% N_2_ for 3, 6, or 12 h, respectively. The cells of the experimental group in each plate were accompanied by the cells of the other plate, which were inoculated at the same time in the complete medium and in the cell culture incubator as a blank control to ensure that the number of cells in the initial well and the culture time were the same. Finally, a 10% CCK-8 reagent was used for the assay, and the absorbance at 450 nm was measured using an enzyme marker to calculate the cell viability.

#### 2.2.12. JC-1 Mitochondrial Membrane Potential Assay

To analyze the changes in mitochondrial membrane potential (MMP; Δ*Ψ*m) induced by OGD treatment at different times, we applied the JC-1 Mitochondrial Membrane Potential Detection Kit (Beyotime, C2006, China) to stain MMP. After incubating the cells with JC-1 solution for 20 min, the MMP was analyzed by fluorescence microscopy, and the ratio of the fluorescence intensity of red (JC-1 aggregates) to that of green (JC-1 monomers) was calculated after processing using teh ImageJ software.

#### 2.2.13. Flow Cytometry Analysis

The apoptosis rate of PC-12 cells was assessed using an AnnexinV-PE/7-AAD Apoptosis Detection Kit (Meilunbio, Dalian, China). Approximately 5 × 10^5^ cells in 500 μL of binding buffer was prepared. After being protected from light, the cells were stained with 5 μL AnnexinV-EGFP and 10 μL PI for 15 min at 4°C. Subsequently, 400 μL of binding buffer was added to the cells, and the apoptosis rate of PC-12 cells was assessed using flow cytometry for normal (AnnexinV−/7-AAD−), early apoptotic (AnnexinV+/7-AAD−), late apoptotic, and necrotic (AnnexinV+/7-AAD+) cells.

### 2.3. Statistical Analysis

All bioinformatics analyses were conducted using the R language package (version 4.3.2), and *p* < 0.05 was deemed statistically significant. Differential gene analysis was performed using the limma package (R v4.3.2) to identify statistically significant expression changes between experimental conditions. Weighted gene co-expression networks were constructed through the WGCNA package (R v4.3.2), enabling the identification of functionally correlated gene modules. Temporal expression patterns were analyzed using the Mfuzz package (R v4.3.2), which implements a fuzzy C-means clustering algorithm to detect phase-specific transcriptional dynamics. KEGG pathway enrichment analysis was conducted via the clusterProfiler package using hypergeometric testing to identify overrepresented biological pathways. Feature selection was implemented using an ensemble approach combining SVM (e1071), kernel-based algorithms (kernlab), and cross-validation frameworks (caret) in R v4.3.2. RF-based feature importance ranking was performed using the randomForest package (R v4.3.2) to identify robust biomarker candidates. Multivariable linear regression was employed to examine correlations between variables.

All experimental data normality was assessed using the Shapiro–Wilk test. Normally distributed data are expressed as mean ± standard deviation (SD) and analyzed as follows: one-way ANOVA for multi-group comparisons, with LSD-t or Dunnett-*t* tests for pairwise comparisons, subsequently followed by Tukey's post hoc test. Non-normally distributed data were evaluated using the Wilcoxon signed-rank test for two-group comparisons or the Kruskal–Wallis H test for multi-group analyses. All analyses were performed in GraphPad Prism 10.1.2, with experimental data representing at least three independent replicates. Statistical significance thresholds were defined as *⁣*^*∗*^*p* < 0.05,*⁣*^*∗∗*^*p* < 0.01, and *⁣*^*∗∗∗*^*p* < 0.001 compared with Sham/control group; *⁣*^#^*p* < 0.05, *⁣*^##^*p* < 0.01, and *⁣*^###^*p* < 0.001 compared with the 3 h group; *⁣*^*Φ*^*p* < 0.05, *⁣*^*ΦΦ*^*p* < 0.01, and *⁣*^*ΦΦΦ*^*p* < 0.001 compared with the 6 h group. *p* > 0.05. Nonsignificant differences (*p* > 0.05) are unmarked.

## 3. Results

### 3.1. Gene Acquisition and Screening

#### 3.1.1. DEG Screening and GSEA Enrichment Analysis

**Figure 1:**
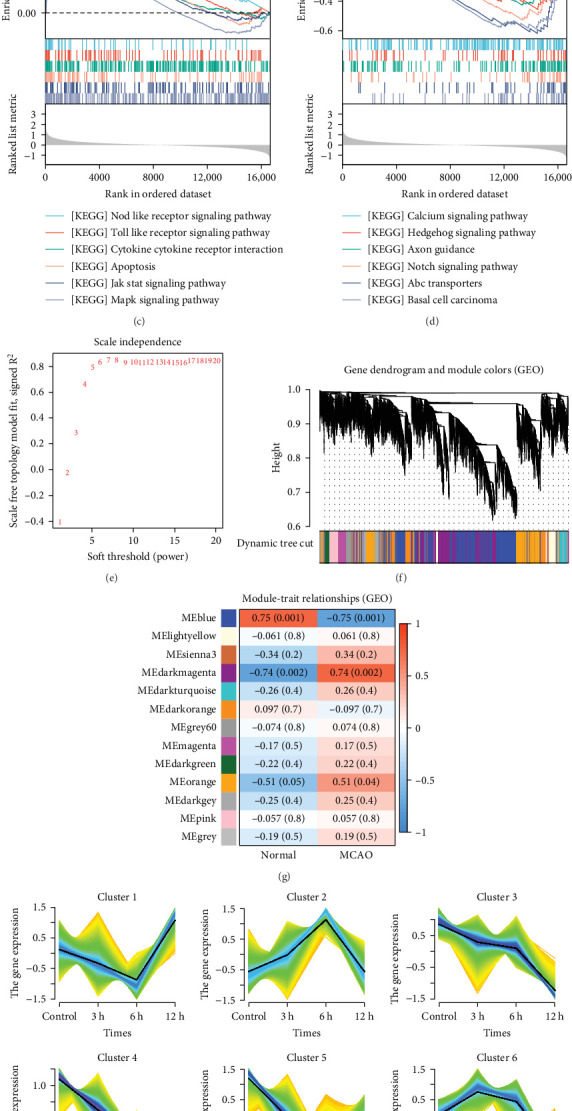
Screening and analysis of differential genes. (a) Box diagram of GSE12216 data homogenization processing. (b) Volcano map of GSE12216 data (red: upregulated; blue: downregulated). (c and d) GSEA_KEGG analysis. (e) The soft threshold power of *β* is 8. (f) Gene co-expression module. (g) Correlation analysis of modules and phenotypes. (h) Analysis of temporal expression patterns.

The data in the GSE212732 dataset of the gene chip were standardized, and the homogenization map was drawn (Figure 1a). The median of the 15 sample data points was basically in a line, indicating that the dataset was of reliable quality and could be analyzed in the next step. DEG screening was performed between the control and MCAO groups using GSE212732. In total, 308 DEGs were identified, comprising 177 upregulated and 131 downregulated genes (Figure 1b). GSEA analysis was conducted on the genes present within the dataset. The GSEA-KEGG results demonstrated that the signaling pathways were activated in the MCAO group were mainly concentrated in the Nod-like receptor signaling pathway, Toll-like receptor (TLR) signaling pathway, cytokine receptor interaction, apoptosis, Jak-Stat signaling pathway, and MAPK signaling pathway (Figure 1c), whereas the downregulated pathways in the MCAO group were mainly concentrated in the calcium signaling pathway, Hedgehog signaling pathway, axon guidance, Notch signaling pathway, ATP-binding cassette transporter, and basal cell carcinoma (Figure 1d).

#### 3.1.2. WGCNA

After data preprocessing, correlation analysis of 11,778 genes was performed, and the soft threshold power of *β* was 8 (Figure 1e) to ensure a scale-free topology model. Gene co-expression modules were then constructed, and these genes were assigned to different modules through a tree diagram (Figure 1f) showing the correlation coefficient between each co-expressed gene module and the stem cell index characteristics. Correlation analysis of the modules and phenotypes revealed a negative correlation between the blue modules and cerebral infarction (*R* = −0.76, *p*=0.001), and the dark magenta and orange modules exhibited a statistically significant positive correlation with cerebral infarction (*R* = 0.74, *p*=0.002; *R* = 0.51, *p*=0.04) (Figure 1g). Therefore, these three models are considered the most important modules related to the disease.

#### 3.1.3. Mfuzz

The Mfuzz package was utilized to investigate the sequence expression patterns of genes at different time points (3, 6, and 12 h) in the GSE212732 dataset. The genes in cluster 4 were time dependent and showed a downward trend. The genes in cluster 9 were time dependent and showed an increasing trend. Therefore, the genes in clusters 4 and 9 were included in the analysis (Figure 1h).

### 3.2. Gene Expression Differences and Effect Mechanisms

#### 3.2.1. GO and KEGG Enrichment Analysis of Significant Disease-Related Genes

**Figure 2:**
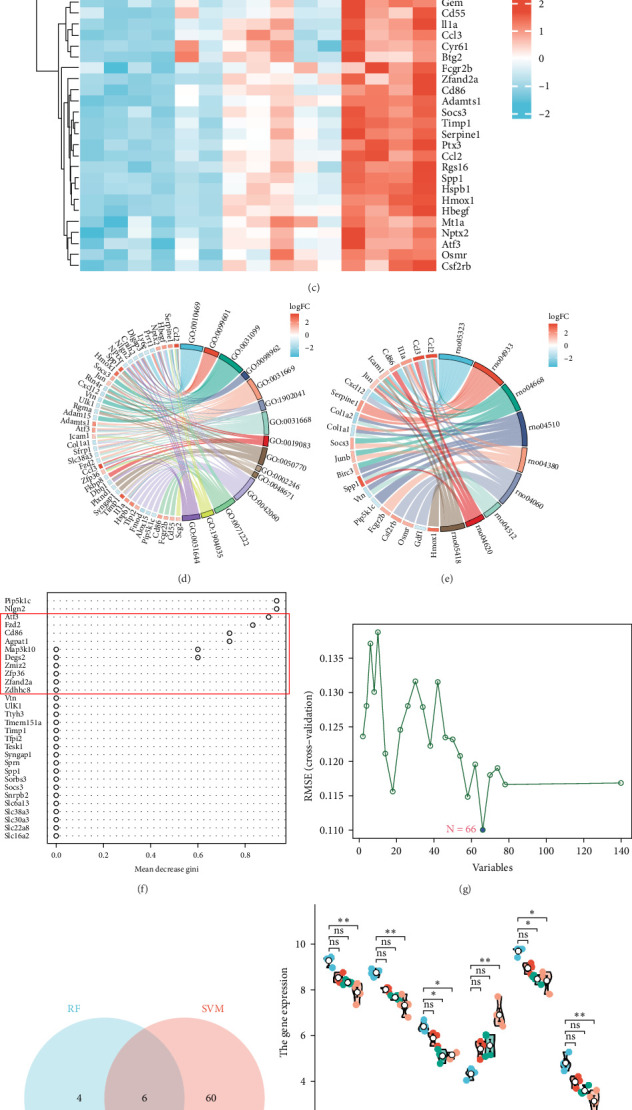
Analysis of disease-related genes and screening of disease-characteristic genes. (a, b) Genes that are significantly associated with the disease. (c) Heat map shows the 25 genes most significantly upregulated and 25 genes most significantly downregulated (red: upregulated; blue: downregulated). (d) GO. (e) KEGG. (f) Ten genes were screened based on the RF algorithm. (g) Sixty-six genes were identified based on he SVM algorithm. (h) Six genes are shared by the two algorithms. (i) Expression of six disease-characteristic genes in the 3, 6, and 12 h cerebral infarction and control group. (j) ROC curve analysis of six disease-characteristic genes.

Genes in the blue, dark magenta, and orange modules, genes in clusters 4 and 9, and disease differential genes in the WGCNA analysis were included in the analysis, and a total of 140 genes were found to be significantly related to disease, of which 40 exhibited a notable increase in expression (Figure 2a), while 100 demonstrated a significant decrease in expression (Figure 2b). The 25 genes exhibiting the most pronounced upregulation and the 25 genes displaying the most significant downregulation were selected and are shown in a heat map (Figure 2c).

GO and KEGG analyses of the 140 genes were performed using ClusterProfiler. Significantly enriched signaling pathways were identified primarily in the context of rheumatoid arthritis, the role of AGE-RAGE signaling pathway in diabetic complications, TNF signaling pathway, focal adhesion, osteoclast differentiation, cytokine–cytokine receptor interaction, extracellular matrix (ECM) receptor interaction, TLR signaling pathway, and fluid shear stress and atherosclerosis signaling pathway (Figure 2d). Biological processes (GO_BP) focused on regulation of signal receptor activity, regulation of neurotransmitter receptor activity, regeneration, regulation of postsynaptic neurotransmitter receptor activity, cellular response to nutrient levels, regulation of exogenous apoptotic signaling pathways through death domain receptors, cellular response to extracellular stimuli, viral transcription, regulation of axonogenesis, and inflammation symptoms associated with wound healing (Figure 2e).

#### 3.2.2. Expression Difference and Receiver Operating Characteristic (ROC) Curve Analysis of Disease-Characteristic Genes

In the RF algorithm, the top ten genes were identified according to their importance (Figure 2f), 66 genes were identified based on the SVM algorithm (Figure 2g), and the Venn package was used to determine the intersection of genes. *Pip5k1c*, *Nlgn2*, *Fzd2*, *Cd86*, *Agpat1*, and *Degs2* were common genes between the two algorithms and were defined as disease-characteristic genes (Figure 2h).

We further analyzed the expression differences of *Pip5k1c*, *Nlgn2*, *Fzd2*, *Cd86*, *Agpat1*, and *Degs2* between the cerebral infarction at 3, 6, and 12 h and the control group. The results demonstrated that, in comparison to the control group, the expression of *Pip5k1c*, *Nlgn2*, *Fzd2*, *Cd86*, *Agpat1*, and *Degs2* showed a downward trend with an increase in the onset time of ischemic stroke, and the decrease was most significant in the MCAO_12 h group (*p* < 0.01). Compared to the control group, the basal expression of the *Cd86* gene showed an increasing trend with an increase in the onset time of ischemic stroke, exhibiting the most pronounced elevation in the MCAO_12 h group (*p* < 0.01) (Figure 2i). ROC curve analysis of *Pip5k1c*, *Nlgn2*, *Fzd2*, *Cd86*, *Agpat1*, and *Degs2* was performed with R package “pROC,” and the area under ROC curve (AUC) was calculated (Figure 2j). The AUC values of *Pip5k1c*, *Nlgn2*, *Fzd2*, *Cd86*, *Agpat1*, and *Degs2* were all 1, suggesting that *Pip5k1c*, *Nlgn2*, *Fzd2*, *Cd86*, *Agpat1*, and *Degs2* have good sensitivity and specificity for the diagnosis of cerebral infarction (Supporting Information S1).

### 3.3. Effect Mechanism Analysis of Disease-Characteristic Genes

**Figure 3:**
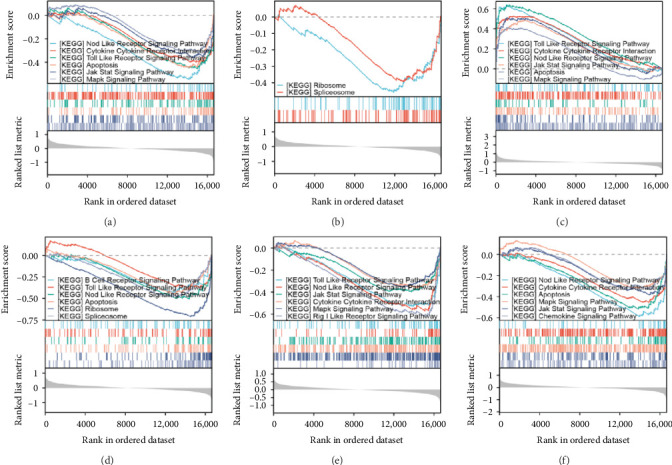
Single-gene GSEA analysis of six disease-characteristic genes in ischemic stroke. (a) Pip5k1c enrichment in Nod-like/Toll-like receptor signaling, cytokine interaction, apoptosis, Jak-Stat, and MAPK pathways. (b) Agpat1 involvement in ribosome and spliceosome. (c) Cd86 in Toll-like/Nod-like receptor signaling, cytokine interaction, Jak-Stat, apoptosis, and MAPK pathways. (d) Degs2 in B cell/Toll-like/Nod-like receptor signaling, apoptosis, ribosome, and spliceosome. (e) Fzd2 in Toll-like/Nod-like receptor signaling, Jak-Stat, cytokine interaction, MAPK, and RIG-I-like pathways. (f) Nlgn2 in Nod-like/cytokine interaction, spoptosis, MAPK, Jak-Stat, and chemokine pathways.

Single-gene GSEA revealed that the identified signature genes (Pip5k1c, Nlgn2, Fzd2, Cd86, Agpat1, and Degs2) converge on neuroinflammatory and apoptotic pathways critical in ischemic stroke pathogenesis. Pip5k1c, Cd86, Degs2, and Fzd2 were prominently associated with TLR and Nod-like receptor signaling pathways (Figure 3a,c,d,e), consistent with the TLR2/TLR4/NF-*κ*B role in driving early-phase (<6 h) neuroinflammation through NF-*κ*B-dependent transcription of IL-1*β*, TNF-*α*, and caspase-1-mediated pyroptosis [11]. Fzd2 was identified as a primary contributor to the Rig-I-like receptor signaling (Figure 3e), which further underscores its dual role in antiviral immunity and mitochondria-dependent apoptosis, paralleling MMP-9-mediated collateral vessel degradation and hemorrhagic transformation risks in acute stroke [17]. The dual association of Degs2 with B cell receptor signaling and ribosome pathways (Figure 3d) may reflect neutrophil-derived immunomodulation, as evidenced by the positive correlation between all-trans 13,14-dihydroretinol (atDR) expression and circulating neutrophil counts, and the selective activation of retinoic acid receptors to exert neuroprotective effects [9]. Intriguingly, Agpat1 was mainly involved in ribosome and spliceosome (Figure 3b), potentially revealing an underappreciated role of post-transcriptional regulation in stroke pathophysiology. This contrasts with established vascular stenosis biomarkers [18] while expanding the scope of metabolic intervention targets. Collectively, these findings position the signature genes within a temporal framework of TLR/NF-*κ*B-driven neuroinflammation and apoptosis, offering mechanistic insights for phase-specific therapeutic interventions.

### 3.4. Verification of the Expression of Disease-Characterizing Genes—In Vivo and In Vitro

To validate the AIS disease signature genes screened by bioinformatics analysis, we measured the mRNA expression levels of brain tissue samples from the Sham group and the 3, 6, and 12 h post-MCAO groups of rats, as well as in cell samples from the PC12 cell control group and the 3, 6, and 12 h post-OGD groups by RT-PCR.

**Figure 4:**
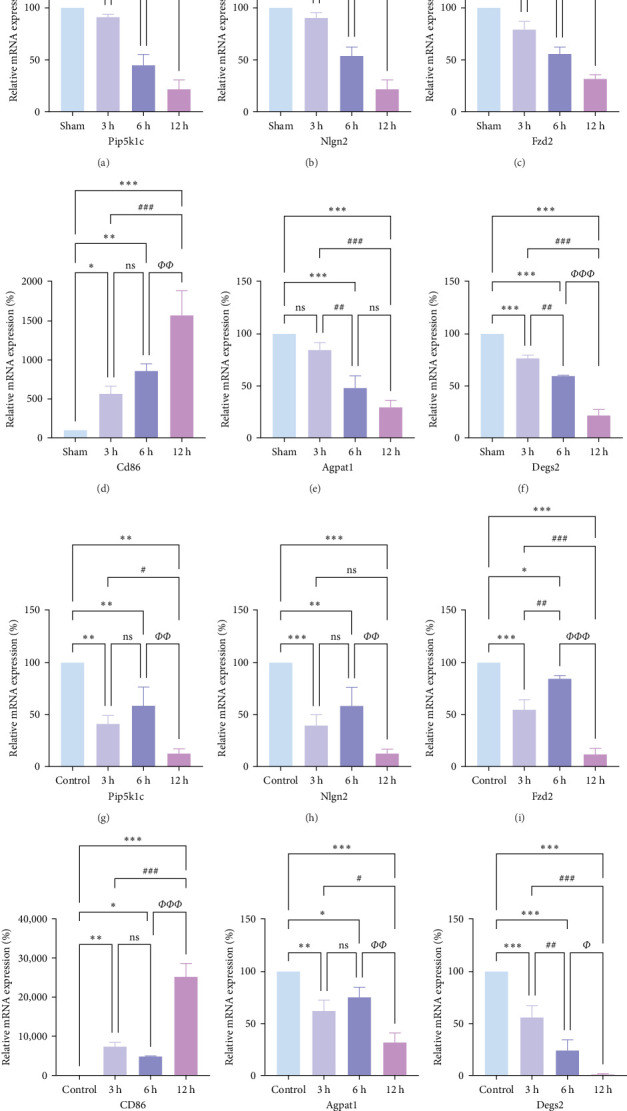
Expression of disease-characterizing genes in vivo and in vitro. (a–f) RT-PCR assay for mRNA expression levels in the brain tissues of rats in the Sham, 3, 6, and 12 h groups (*n* = 5). (g–l) RT-PCR method was used to detect the mRNA expression levels of PC-12 cells in the control, 3h, 6h, and 12 h groups (*n* = 5). *p* > 0.05, not statistically significant. *⁣*^*∗*^*p* < 0.05, *⁣*^*∗∗*^*p* < 0.01, and *⁣*^*∗∗∗*^*p* < 0.001 compared with Sham/control group; *⁣*^#^*p* < 0.05, *⁣*^##^*p* < 0.01, and *⁣*^###^*p* < 0.01 compared with 3 h group; *⁣*^*Φ*^*p* < 0.05, *⁣*^*ΦΦ*^*p* < 0.01, and *⁣*^*ΦΦΦ*^*p* < 0.001 compared with the 6 h group indicated statistically significant differences.

In rats, although mRNA expression was no statistically significant difference in the Sham group compared to the 3 h (*Pipk1c*, *Nlgn2*, and *Agpat1*) and the 6 h compared to the 12 h group (*Agpat1*) (Figure 4a,b,e), the mRNA expression of *Pip5k1c*, *Nlgn2*, *Fzd2*, *Agpat1*, and *Degs2* showed a decreasing trend with infarct extension, with the greatest difference in the 12 h group compared to the Sham group (Figure 4). Although no statistically significant difference was observed in Cd86 mRNA expression between the 3 and 6 h groups (Figure 4d), an increasing trend was evident with increasing infarction time and the difference was most significant in the 12 h group compared with the Sham group. This finding aligns with the results of the bioinformatics analysis (Supporting Information S2).

In PC12 cells, there was a notable decrease in the expression of Pip5k1c, Nlgn2, Fzd2, Agpat1, and Degs2 mRNA, while Cd86 mRNA expression exhibited an increase in all experimental groups when compared to the control group. The distinction in mRNA expression of the aforementioned genes was most evident in the 12 h group (Figure 4). It is noteworthy that the mRNA expression of *Pip5k1c*, *Nlgn2*, *Fzd2*, and *Agpat1* in the 3 h group was observed to be lower than that in the 6 h group (Figure 4g,h,k), while *Cd86* mRNA expression in the 3 h group was found to be higher than that in the 6 h group (Figure 4j). Some of the characterized genes did not show progressively significant mRNA expression differences with the prolongation of the intervention time. Instead, there was a fluctuating trend in mRNA expression levels that did not align with the findings of the bioinformatics analysis. (Supporting Information S3).

### 3.5. Significant Signs of Brain Tissue Infarction and Neurological Deficits in Middle Cerebral Artery Occlusion (MCAO) Rats

**Figure 5:**
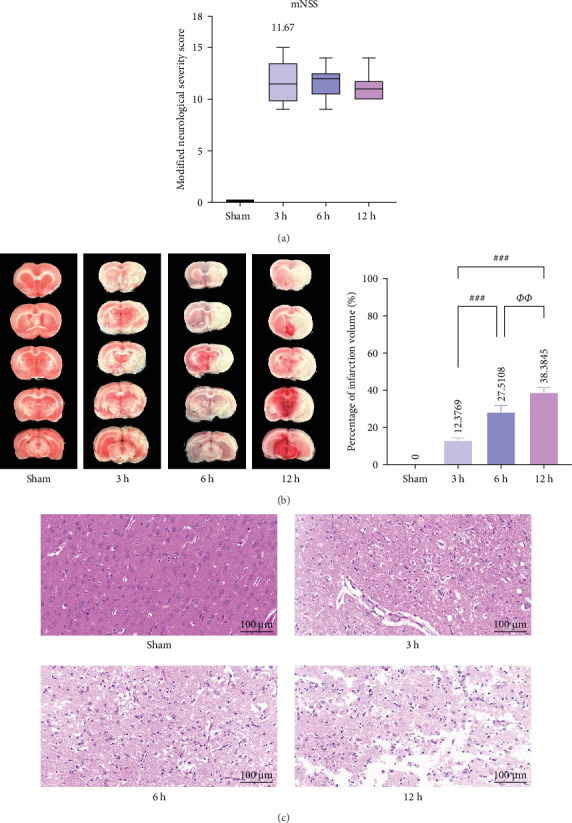
Analysis of nerve deficit scores and degree of infarction in rats. (a) Neurological deficit score (*n* = 5). (b) Quantification of TTC-stained cerebral infarct volume as a percentage of hemispheric volume (*n* = 3). *p* > 0.05, not statistically significant. *⁣*^*∗*^*p* < 0.05, *⁣*^*∗∗*^*p* < 0.01, and *⁣*^*∗∗∗*^*p* < 0.001 compared with Sham/control group; *⁣*^#^*p* < 0.05, *⁣*^##^*p* < 0.01, and *⁣*^###^*p* < 0.01 compared with 3 h group; *⁣*^*Φ*^*p* < 0.05, *⁣*^*ΦΦ*^*p* < 0.01, and *⁣*^*ΦΦΦ*^*p* < 0.001 compared with the 6 h group indicated statistically significant differences. (c) Representative H&E staining photographs of rats in different groups.

To evaluate the middle cerebral artery occlusion (MCAO) model, we assessed the degree of infarction and the mNSS score in rats. We evaluated rats at different time points (3, 6, and 12 h); the mNSS was zero in the Sham group, while the mean mNSS score after modeling was 11.67, indicating the successful establishment of the MCAO model (Figure 5a).

Immediately after the mNSS measurement, we evaluated the focal infarct volume in the rat brain. Infarct foci were formed in all experimental groups (white area), and the infarct volume was significantly different between the groups (*p* < 0.01); however, the infarct foci were not obvious at 3 h after MCAO (mean = 12.3769%). (Figure 5b). H&E staining showed that neurons in the cerebral cortex of the Sham group exhibited orderly arrangement and structural normality, with no evidence of inflammatory cell infiltration. A small amount of neuronal damage was observed in the 3 h group and only a few fluid vesicles were seen around the cells, but significant neuronal damage and obvious vacuoles were observed in the 6 h group. In the 12 h group, most of the cerebral cortex was liquefied and necrotic, neurons were heavily damaged, gliosis was observed, and a considerable number of vesicles were observed in the surrounding area. The degree of brain tissue damage changed significantly as the infarction time prolonged (Figure 5c).

### 3.6. In Vivo Observation of Temporal Trends in Neuroinflammation During the Hyperacute Phase of AIS

**Figure 6:**
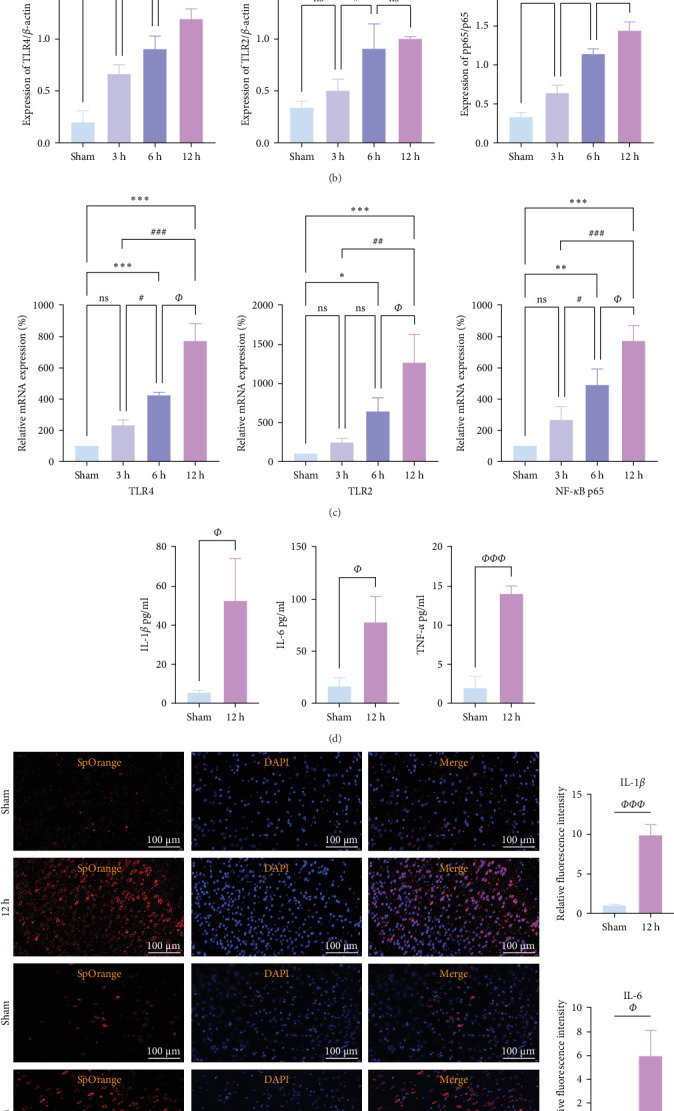
TLR2/TLR4/NF-*κ*B signaling pathway regulates neuroinflammatory responses in the hyperacute phase of MCAO rats. (a, b) Western blot detection of protein expression of TLR2, TLR4, NF-*κ*Bp65, and phosphorylated NF-*κ*Bp65 in rat brain tissues (*n* = 3). (c) RT-PCR detection of mRNA expression of TLR4, TLR2, and NF-*κ*B in rat brain tissues (*n* = 5). (d) Enzyme-linked immunosorbent assay was used to detect the expression levels of IL-1*β*, IL-6, and TNF-*α* in the centrifuged supernatant of rat brain tissue (*n* = 5). (e) The immunofluorescence technique used to detect the distribution and expression levels of IL-6, IL-1*β*, and TNF-*α* in rat brain tissue (*n* = 5). *p* > 0.05, not statistically significant; *⁣*^*∗*^*p* < 0.05, *⁣*^*∗∗*^*p* < 0.01, and *⁣*^*∗∗∗*^*p* < 0.001 compared with the Sham/control group; *⁣*^#^*p* < 0.05, *⁣*^##^*p* < 0.01, and *⁣*^###^*p* < 0.01 compared with the 3 h group; *⁣*^*Φ*^*p* < 0.05, *⁣*^*ΦΦ*^*p* < 0.01, and *⁣*^*ΦΦΦ*^*p* < 0.001 compared with the 6 h group indicated statistically significant differences.

We focused on whether the neuroinflammatory response in the hyperacute phase of AIS is closely related to characteristic gene effector mechanisms, especially the TLR signaling pathway. Taking the TLR2/TLR4/NF-*κ*B pathway as the entry point, we found that the mRNA and protein expression of TLR2, TLR4, and NF-*κ*B were progressively upregulated with the prolongation of infarction time (Figure 6), and a significant difference was observed in the 12 h compared with the Sham group (*⁣*^*∗∗*^*p* < 0.01). Even though the protein expression of non-phosphorylated NF-*κ*B did not show significant inter-group changes, the ratio of phosphorylated NF-*κ*B to non-phosphorylated NF-*κ*B protein showed significant differences compared with the control group for all periods (Figure 6a,b). Subsequently, we employed the ELISA and IF methods to observe the difference in the expression of inflammatory factors IL-1*β*, IL-6, and TNF-*α* in the downstream cascade of the TLR2/TLR4/NF-*κ*B pathway between the Sham and 12 h group more intuitively, and the results revealed a significant increase in expression in the 12 h group (Figure 6d,e). The TLR2/TLR4/NF-*κ*B pathway exhibited a pronounced activation in the hyperacute phase of AIS (Figure 6d,e), with a discernible correlation between its expression and the prolongation of infarction time.

### 3.7. In Vivo Observation of Neuronal Apoptosis Dynamics in the Hyperacute Phase of AIS

In AIS, the neuroinflammatory response is closely related to neuronal apoptosis, and NF-*κ*B signaling mediated by TLR2 and TLR4 through the MyD88-dependent pathway can activate apoptosis [19] in response to changes in the expression of proapoptotic markers (e.g., Bax, Caspase3) and the anti-apoptotic protein Bcl-2 [20]. The high frequency of the TLR signaling pathway and apoptosis mechanism in the GESA analysis results supported this conclusion, and we observed changes in neuronal cell injury and apoptosis in vivo.

**Figure 7:**
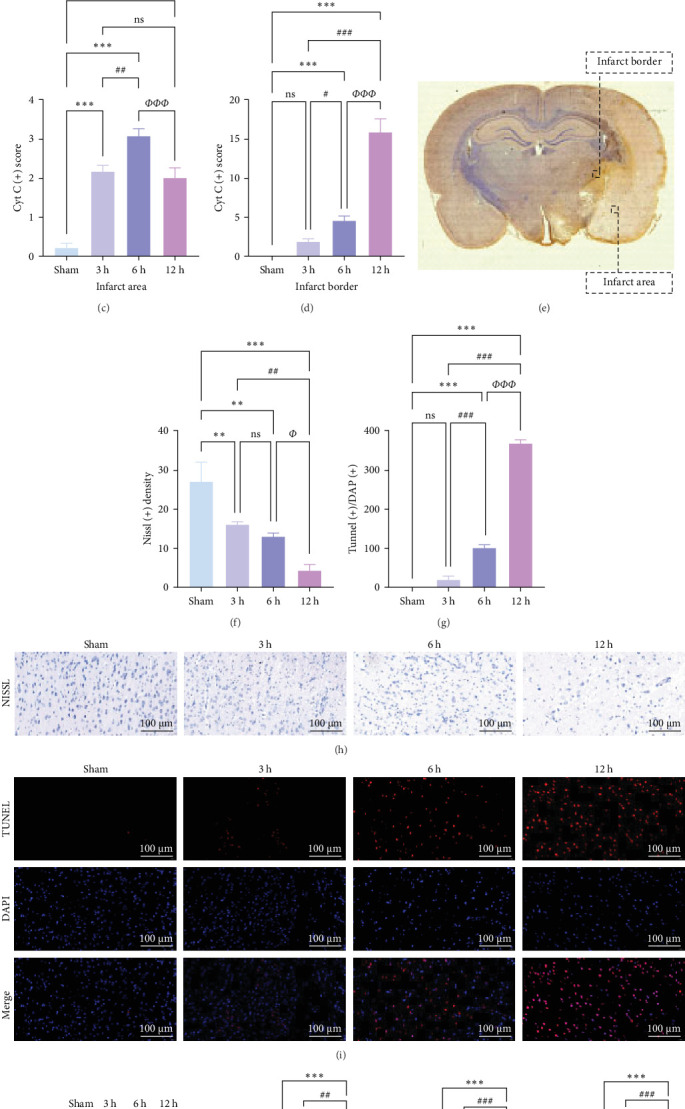
Dynamic changes of apoptosis in brain tissues of rats with MCAO in the hyperacute phase (a, c) are representative images and quantitative analyses of the infarcted area in each group of rats, (*n* = 5), (b, d) are representative images and quantitative analyses of the infarct border in each group of rats, (*n* = 5), (e) is a representative image of Cyt-c immunohistochemical staining, (f, h) are representative images and quantitative analyses of Nissl staining (*n* = 5), (g, i) are representative images and quantitative analyses of TUNEL staining (*n* = 5), (j, k) show western blot assay for protein expression of Bax, Bcl-2, cl-Caspase-3, and Cyt-c in rat brain tissue. *p* > 0.05, not statistically significant; *⁣*^*∗*^*p* < 0.05, *⁣*^*∗∗*^*p* < 0.01, and *⁣*^*∗∗∗*^*p* < 0.001 compared with Sham/control group; *⁣*^#^*p* < 0.05, *⁣*^##^*p* < 0.01, and *⁣*^###^*p* < 0.01 compared with 3 h group; *⁣*^*Φ*^*p* < 0.05, *⁣*^*ΦΦ*^*p* < 0.01, and *⁣*^*ΦΦΦ*^*p* < 0.001 compared with 6 h group indicated statistically significant differences.

Cytochrome c (Cyt-c) is mainly present in the mitochondria during normal cellular activities. When the mitochondrial apoptotic pathway is activated, Cyt-c is released into the cytoplasm to activate cysteine proteases (e.g., Caspase 3) leading to apoptosis. Immunohistochemical staining of Cyt-c in rat brain tissue (Figure 7a,c,e) revealed that the brain tissue of the Sham group had a dense structure, and the neurons contained little Cyt-c, which was arranged in the form of regular granules related to its confined distribution in the mitochondria of normal cells. Cyt-c exhibited distinct spatiotemporal patterns between infarct zones. In the core region, a triphasic “elevation-diffusion-depletion” trajectory was observed. Compared with the Sham group, with the prolongation of infarction time, Cyt-c expression in the infarct core showed an increasing trend at 3 and 6 h and decreased at 12 h. The distribution of Cyt-c became disordered with the change in neuronal morphology, and there was obvious disordered distribution and abundant brown granule staining in the 6 h group. However, in the 12 h group, the distribution of Cyt-c did not increase because of more severe changes in neuronal morphology but rather decreased.

For this reason, we found that in the infarct border (Figure 7b,d,e), although the difference in Cyt-c expression in the 3 h group was not statistically significant, there was an abnormal delayed elevation of Cyt-c expression in the 12 h group (*p* < 0.001), which showed intense dark brown granules distributed in the form of lines and clusters. In the present study, the decrease of Cyt-c expression in the infarct core after 12 h was related to the decrease of post-infarct neuron number in this area and the release of Cyt-c from the apoptotic cells, which had already occurred. In contrast, the abnormal increase in Cyt-c expression in the infarct margin after 12 h was related to further expansion of the ischemic penumbra and an increase in neuronal cells undergoing apoptosis, especially the mitochondrial apoptosis pathway. In addition, the morphology of the brain tissue changed after the infarction and became loose and porous. With prolonged infarction, the elevated expression of Cyt-c in the infarct border may be related to the leakage of Cyt-c to the infarct border after the infarction of neurons in the core area of the infarction.

The results of Nissl staining (Figure 7f,h) indicated that the neurons in the Sham group were neatly arranged and densely packed. The cytosol was full, the coloring was darker, the nuclei were large and round, and there were numerous Nissl bodies. In the 3 h group, there was a decrease in the number of nitrosomes (*⁣*^*∗∗*^*p* < 0.01) and a small number of vacuoles, but the number of neuronal cells was still high, the cytosol was relatively full, and the morphological changes were not obvious. In the 6 h group, the number of neurons decreased significantly and the cytosol gradually atrophied, although the quantitative data were less significant than those of the 3 h group (*p* > 0.05). In contrast, in the 12 h group, obvious morphological changes were detected in the infarcted area of the brain, the neurons were the scarcest, the nuclei of the cells were atrophied and lightly colored, the cytosol was atrophied and vacuolated, and the Nissl bodies were rare. TUNEL staining (Figure 7g,i) showed that in the hyperacute phase, apoptosis of rat brain tissue increased with the prolongation of infarction time, and the number of apoptotic cells increased significantly after 12 h (*⁣*^*∗∗∗*^*p* < 0.001).

WB results demonstrated that, in comparison to the Sham group, the protein expression ratio of Bax/Bcl2 in the experimental group exhibited a gradual increase, accompanied by a similar trend in the expression of Caspase3 and Cyt-c (Figure 7j,k) (Supporting Information S4). Therefore, neuronal apoptosis gradually increased with infarction time, and the differences between the experimental groups were evident. These trajectories strongly correlated with TLR2/TLR4/NF-*κ*B pathway activation kinetics, suggesting synchronized regulation of inflammatory and apoptotic cascades.

### 3.8. Temporal Trends of Neuroinflammation in the Hyperacute Phase of AIS Observed In Vitro

**Figure 8:**
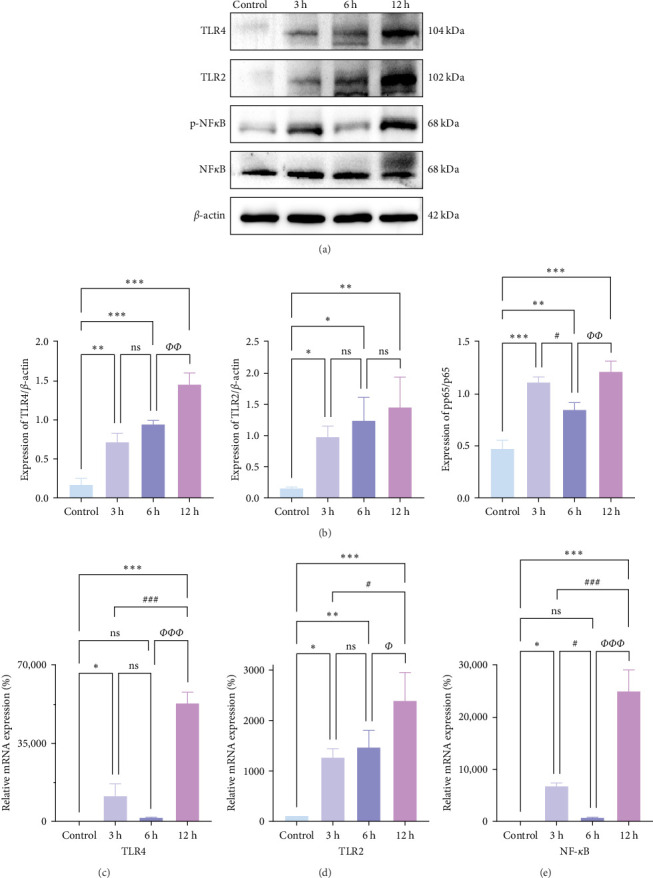
Observation of temporal trends in inflammation of PC-12 cells. (a, b) Western blot detection of protein expression of TLR2, TLR4, non-phosphorylated NF-*κ*Bp65, and phosphorylated NF-*κ*Bp65 in PC-12 cells (*n* = 3). (c–e) RT-PCR assay to detect mRNA expression of TLR4, TLR2, and NF-*κ*B in PC-12 cells (*n* = 5). *p* > 0.05, not statistically significant; *⁣*^*∗*^*p* < 0.05, *⁣*^*∗∗*^*p* < 0.01, and *⁣*^*∗∗∗*^*p* < 0.001 compared with the Sham/control group; *⁣*^#^*p* < 0.05, *⁣*^##^*p* < 0.01, and *⁣*^###^*p* < 0.01 compared with the 3 h group; and *⁣*^*Φ*^*p* < 0.05, *⁣*^*ΦΦ*^*p* < 0.01, and *⁣*^*ΦΦΦ*^*p* < 0.001 compared with the 6 h group for statistically significant differences.

We further observed the temporal trend of the TLR2/TLR4/NF-*κ*B signaling pathway regulating neuroinflammation in the hyperacute phase of AIS in vitro. The results demonstrated that the expression of TLR2 increased with the prolongation of the intervention time (Figure 8a,b,d; *⁣*^*∗*^*p* < 0.05) (Supporting Information S4); however, no gradual increase was observed in the expression of TLR4 and NF-*κ*B. Even though the differences between TLR4 and NF-*κ*B were significant in the 12 h group compared with the control group (*⁣*^*∗∗∗*^*p* < 0.001), the mRNA expression of TLR4 and NF-*κ*B, and the ratio of phosphorylated NF-*κ*B to nonphosphorylated NF-*κ*B protein expression were lower in the 6 h group compared with the 3 h group (Figure 8a,b,c,e). Suggests the TLR2/TLR4/NF-*κ*B signaling pathway post-transcriptional dynamic feedback related to presence of the mechanisms.

### 3.9. In Vitro, Apoptosis in the Hyperacute Phase of AIS Showed a Fluctuating Increase With Prolonged Infarction Time

**Figure 9:**
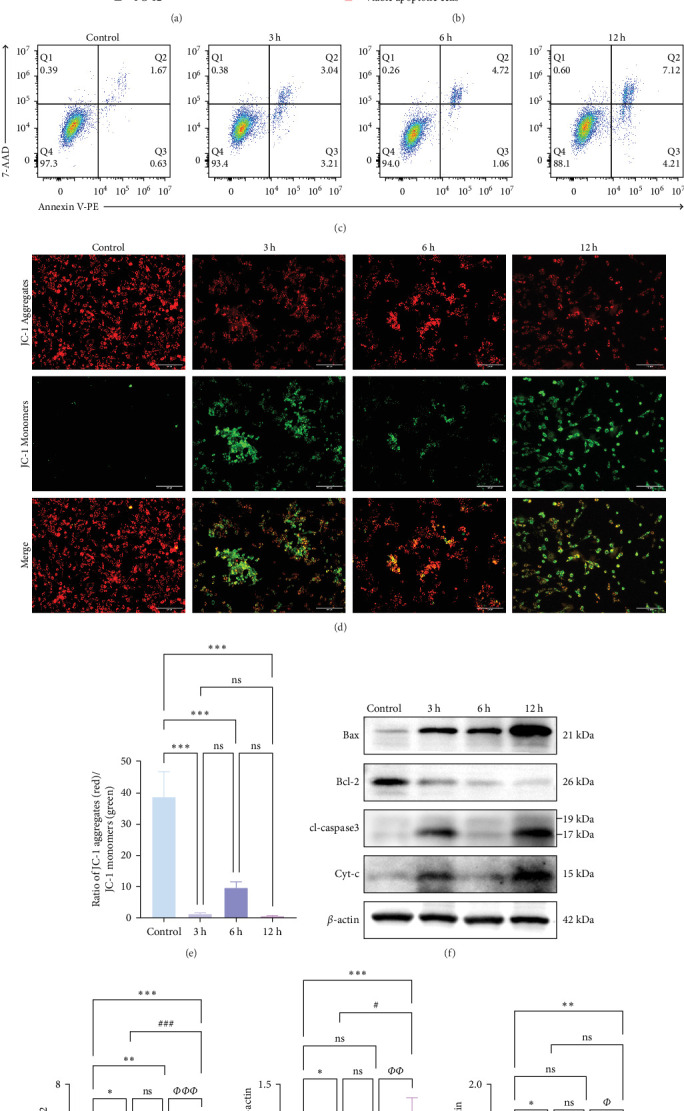
PC-12 cells showed a cliff-like apoptosis time trend. (a) CCK-8 was used to determine the viability of PC-12 cells (*n* = 5). (b, c) Flow cytometry was used to detect early apoptosis, late apoptosis, and overall apoptosis of PC-12 cells (*n* = 5). (d, e) Representative images and quantitative analysis of PC-12 cells subjected to JC-1 staining, (*n* = 5). (f, g) Western blot was used to detect protein expression of PC-12 cells of protein expression of Bax, Bcl-2, cl-Caspase-3, and cytochrome C (*n* = 3). *p* > 0.05, not statistically significant; *⁣*^*∗*^*p* < 0.05, *⁣*^*∗∗*^*p* < 0.01, and *⁣*^*∗∗∗*^*p* < 0.001 compared with the Sham/control group; *⁣*^#^*p* < 0.05, *⁣*^##^*p* < 0.01, and *⁣*^###^*p* < 0.01 compared with the 3 h group; *⁣*^*Φ*^*p* < 0.05, *⁣*^*ΦΦ*^*p* < 0.01, and *⁣*^*ΦΦΦ*^*p* < 0.001 compared with the 6 h group indicated statistically significant differences.

We first detected the viability of cells in each group. The cells viability gradually decreased as the intervention time extended, dropping to less than 50% at 12 h (Figure 9a).

In the flow cytometry assay (Figure 9b,c), the proportion of total apoptotic cells and late apoptotic cells increased gradually with prolonged intervention time. Nevertheless, the proportion of early apoptotic cells was minimal and not statistically different from that of the control group at 6 h, whereas the maximum values of the proportion of early apoptotic cells were observed at 3 and 12 h (*⁣*^*∗*^*p* < 0.05). We examined MMP changes to further explore early apoptotic patterns during the hyperacute phase of AIS. It is noteworthy that despite the pronounced disparities between the experimental groups and the control group (*p* < 0.001), there was no discernible trend of gradual decrease with the prolongation of infarction time among the experimental groups (*p* > 0.05). Nevertheless, as with the findings of the flow cytometry, there was a notable reduction in the MMP in the 3 and 12 h group (*p* < 0.001), while in the 6 h group, the MMP exhibited a less pronounced decline compared to the other experimental groups (Figure 9d,e).

Western blotting (WB) results (Figure 9f,g) (Supporting Information S4) showed that the protein expression ratio of Bax/Bcl2 exhibited a gradual increase with the prolongation of infarction time (*⁣*^*∗*^*p* < 0.05), whereas the protein expression of cl-Caspase3 and Cyt-c showed significant increases at 3 and 12 h, respectively, in comparison to the control group (*p* < 0.05), and no statistically significant trend was observed in the 6 h group (*p* > 0.05).

This was analogous to the mRNA expression trend of TLR4 and NF-*κ*B, as well as the protein expression trend of the ratio of phosphorylated NF-*κ*B to non-phosphorylated NF-*κ*B (Figure 8b,c,e). These findings suggest that apoptosis occurring in AIS may be regulated by TLR4/NF-*κ*B, which implies that the TLRs pathway may mediate apoptotic oscillations through regulation of the mitochondrial apoptotic pathway. Moreover, they indicate that apoptosis after cerebral infarction may not gradually increase steadily with prolongation of infarction. Instead, it exhibits a fluctuating trend of apoptosis, with peaks in early apoptosis at the 3 and 12 h periods and a trough at the 6 h.

## 4. Discussion

There is an urgent clinical need to overcome the limitations of biomarkers and therapeutic interventions in hyperacute AIS management. Current clinical challenges stem from three critical gaps: (1) Temporal mismatch between treatment windows and biomarker availability. While advanced imaging techniques (e.g., CTP, DWI-FLAIR mismatch) have expanded the thrombolytic time window to 24 h [21], ~30% of patients still miss the 4.5-hour therapeutic window due to delayed biomarker detection [22]. Fda-approved biomarkers such as S100*β* take 8 h to reach the diagnostic threshold [23], which creates a critical 3.5-hour “molecular blind spot.” (2) Therapeutic heterogeneity and limitations of traditional biomarkers. Traditional biomarkers predominantly detect secondary injury [24], rather than the initial event of the ischemic cascade. Despite mechanical thrombectomy's efficacy in large vessel occlusion (LVO), up to 40% of patients with medium vessel occlusion (MeVO) fail to achieve functional independence even after successful recanalization. This heterogeneity stems from the inability of current biomarkers to identify early ischemic pathophysiology that predicts reperfusion injury. Finding biomarkers that can stratify patients in conjunction with current disease status is extremely important [25]. For example, TRACE-III demonstrated that TNK thrombolysis improved outcomes in non-thrombectomy candidates [26], while TIMELESS showed no benefit when combined with MT due to delayed biomarker-guided selection [27]. (3) Limitations of neuroprotective agents: While multi-target drugs like edaravone dexborneol show promise in reducing disability rates [28], most single-mechanism neuroprotectants fail in Phase III trials due to narrow therapeutic targets and inadequate consideration of blood-brain barrier dynamics. These gaps underscore the urgency of developing biomarkers that target the hyperacute phase of AIS. Our study addresses these gaps by identifying hyperacute-phase signature genes that map to the ischemic cascade initiation.

Based on the bioinformatics signature results, we identified the disease signature genes Pip5k1c, Nlgn2, Fzd2, Cd86, Agpat1, and Degs2 specific to the hyperacute phase of AIS. The temporal precision of our study encompassed the thrombolytic therapy window (0–6 h). Moreover, the selected feature genes were centered on the crucial “ischemic initiation” stage. This approach enabled the capture of the onset of neuroinflammation and apoptosis, as opposed to the outcome, which differed from traditional markers that reflect end-stage injury. Our study aimed to bridge the gap in biomarkers for the hyperacute stage of AIS. *Pip5k1c* (phosphatidylinositol-4-phosphate5-kinase type1gamma) is a protein-coding gene that is highly expressed in brain [29], is closely associated with neuronal and nervous system development [30]. Deletion of *Pip5k1c* triggers integrin-FAK signaling pathway dysregulation, leading to ECM degradation via MMP-9 upregulation and caspase-3-mediated apoptosis [31] through Bcl-2/Bax imbalance [32]. *Nlgn2* (Neuroligin-2) is a gene associated with neurodevelopmental disorders [33]. Overexpression of *Nlgn2* in schizophrenic neurons rescues synaptic point defects [34]. *FZD2* (Frizzled Class Receptor 2) is a key regulator of cellular processes [35]. Additionally, it plays a role in the DNA damage repair process. FZD2 plays a role in the regulation of classical Wnt and TLR signaling pathways [36], which are associated with inflammatory and vascular pathological processes [37]. *CD86* (also known as B7-2) is an important co-stimulatory molecule expressed on the surface of antigen-presenting cells (APCs), affects the interaction of TLRs with APCs, and is closely related to T-cell activation, proliferation, and immune function. Changes in CD86 may lead to immune cell dysfunction, activate the apoptosis in autoreactive T cells, and trigger subsequent systemic inflammatory responses. This represents a dual immune-regulatory mechanism, which explains its potential as a biomarker [39]. Some studies have found an association between the CD86 gene and several systemic autoimmune disorders, including multiple sclerosis [40], rheumatoid arthritis [41], and Graves' ophthalmopathy [42]. This may suggest that CD86 is closely related to the neuroinflammatory response after AIS, especially the perivascular inflammatory niches generated by the disruption of the spatial coupling of CD86-TLR2. *Agpat1* (1-acylglycerol-3-phosphate O-acyltransferase 1) is a protein-coding gene associated with glycerol ester biosynthesis and energy metabolism [43], and its related disorders present metabolic, developmental, and especially neuronal developmental and functional changes as a result of disruption of lipid homeostasis [44]. *Degs2* (delta4-desaturase, sphingolipid2) encodes a bifunctional enzyme that plays a role in the biosynthesis of phytosphingolipids in human skin and tissues containing phytosphingolipids, and is expressed in the brain and various peripheral tissues [45]. Previous studies have demonstrated that *Degs2* expression is reduced in patients with major depression [46]. Moreover, several investigations have indicated that the DEGS2-Bcl-2 complex regulates ceramide-induced cytochrome-c release, thereby positioning it at the nexus of sphingolipid signaling and apoptotic cascades in CNS injuries [46, 47]. However, further research is required to elucidate the precise mechanisms involved.

The mRNA expression trend of the characterized genes was similar to the trend predicted by biosignature analysis, which initially verified its credibility and feasibility. The characterized genes should be analyzed together with the related disease pathogenesis to help further define disease biomarkers. We individually carried out weighted enrichment statistics on the disease signature genes by employing the GSEA method. The outcomes demonstrated the frequent manifestation of the TLR signaling pathway and apoptosis. This serves as a crucial bridge connecting the early molecular events with the initiation of ischemia and pathological phenotypes. Therefore, in the subsequent experiments, we mainly observed the specific mechanisms of these two pathological processes in the hyperacute phase of AIS.

TLRs serve as essential components of the innate immune system. These receptors play pivotal roles in both inflammatory processes and tumorigenesis [48]. Specifically, TLR2 [49] and TLR4 [50] are closely associated with neuroinflammatory responses in AIS and are partially expressed in cerebrovascular endothelial cells, oligodendrocytes, and neurons [51]. The TLRs signaling pathway is mainly activated by the myeloid differentiation factor88 (MyD88)-dependent pathway and the Toll/IL-1receptor (TIR)-domain-containing adapter inducing interferon-*β* (TRIF)-dependent pathway [52]. TRIF can, in turn, cause phosphorylation of IFN-*β* and induces transcription of other genes. It also directly binds to TNF receptor-associated factor 6 (TRAF6) and receptor-interacting protein (RIP), ultimately activating NF-*κ*B and AP-1. The activation of NF-*κ*B leads to the activation of inflammatory factors (e.g., TNF-*α*, IL-1*β*, IL-6, IL-8, and NO) and adhesion molecules, promoting inflammation. TLR2 and TLR4 function in AIS mainly through the pathways described above [53] and are independently correlated with the expression of various types of inflammatory mediators and poor prognosis. Additionally, TLR2 and TLR4 act as independent mutants to increase vascular permeability after AIS, aggravating inflammatory injury after cerebral ischemia [54]. Therefore, this study specifically focused on the TLR2/TLR4/NF-*κ*B signaling pathway as the research target, investigating its temporal dynamics in mediating neuroinflammatory responses during the hyperacute phase of AIS. Our multi-timepoint analysis revealed a linearly progressive intensification pattern of neuroinflammation regulated by this pathway.

Apoptosis represents one of the most prevalent forms of cell death following cerebral ischemia. In the hyperacute phase of AIS, apoptosis commences early in the neurons of brain tissue and exerts a significant impact on the outcome of AIS. The endogenous pathway, which is closely related to AIS, is activated by the apoptotic protein Bid, which induces the activation of the apoptotic protein Bcl-2 antagonist, Bak, and the Bcl-2 binding protein, Bax, which form aggregates to cause a decrease in MMP and the release of apoptosis-critical proteins, thereby activating apoptosis in the mitochondrial pathway. A key aspect of apoptosis involves proapoptotic proteins (Bid, Bax) [55] and antiapoptotic proteins (Bcl-2 and Bcl-x) [56], and a balance between the promoter and effector cysteinyl aspartate-specific proteinases (caspases). Upon receiving death signals, apoptosis-related proteins translocate to the mitochondria, altering the MMP and releasing the pro-apoptotic factor Cyt-c from the mitochondria [57]. The released Cyt-c from mitochondria bind to various proteins in the cytoplasm, forming apoptotic bodies and triggering several cellular cascades, especially the caspase cascade, which generates Caspase-3, which ultimately leads to apoptotic cell death. The present study aimed to elucidate the combined action of Bcl-2 and Bax, and the caspase cascade at multiple time points to observe the pattern of apoptosis during the hyperacute phase of AIS.

In addition, the TLRs itself induce apoptosis [38]. NF-*κ*B activated by TLR4 through the MyD88-dependent pathway increases the expression of cysteinyl aspartate-specific proteinase11 (caspase-11), which is immediately followed by the activation of Caspase-3, which induces apoptosis [19]. Phosphorylation of NF-*κ*B leads to the release of several inflammatory factors (e.g., TNF-*α*, IL-6, and IL-1*β*) also induces apoptosis [58, 59]. Moreover, TLR2 regulates the expression of Caspase-8, partially promoting apoptosis [20]. The TLRs signaling pathway and apoptosis appeared in near-synchronous high frequency in the disease signature gene effector mechanisms analyzed by GSEA. In vivo, the neuroinflammatory response was highly synchronized with neuronal apoptosis, and in vitro, the trend of NF-*κ*B expression was similar to that of the caspase cascade apoptosis. This suggests that the TLRs signaling pathway is also closely linked to the apoptotic mechanism in the hyperacute phase of AIS and supports the possibility that the biomarkers screened in this study may serve as potential therapeutic targets for the hyperacute phase of AIS.

Influenced by the concept of “time is brain” in stroke management, it has become a stereotype that brain tissue damage is more severe with prolonged infarction, which is extremely important in emphasizing early diagnosis and treatment of stroke. Our experiment validated and observed the regulatory pattern of neuroinflammation. TLR2 and TLR4 prompt the rapid activation of NF-*κ*B via the MyD88-dependent pathway, inducing the release of proinflammatory factors such as TNF-*α* and IL-6. This process establishes a robust positive feedback that leads to the dynamic exacerbation of inflammation during the hyperacute phase of AIS. However, it remains unclear whether each mechanism of pathological damage in AIS continues to increase with prolonged infarction. In evaluating the mechanisms of apoptosis, we found that the apoptotic mechanisms occurring in vitro were not progressive, and that there were nonlinear changes. Upon initial examination of the the mRNA expression of genes characterized by the disease, it was observed that the expression of certain genes exhibited a lower level in the 6 h group compared to the 3 h group. Notably, in vitro experiments revealed a biphasic fluctuation pattern in apoptotic indicators (Bax/Bcl-2 ratio, Caspase-3, and Cyt-c) at 3 and 12 h post-AIS, mirroring the dual-peak trend observed in characteristic gene expression profiles. This temporal dissociation from the monotonically progressive neuroinflammation suggests potential compensatory mechanisms buffering apoptotic progression. Specifically, early apoptotic peaks at 3 and 12 h were separated by an unexpected trough at 6 h. We hypothesize that Nlgn2 downregulation may activate the Notch-Hes1 pathway, thereby attenuating TLR-mediated apoptosis through NF-*κ*B suppression [60]. Concurrently, Pip5k1c-mediated PI (4,5) P2 synthesis could modulate autophagic flux to eliminate damaged mitochondria and mitigate cGAS-STING hyperactivation [61]. These coordinated molecular adaptations may collectively shape the nonlinear apoptotic evolution during the AIS hyperacute phase. In turn, this biphasic apoptotic pattern, which is shaped by these adaptations, carries profound therapeutic implications and demands phase-targeted interventions: (1) Early window (0–3 h): TLR4/CD86 blockade using TAK-242 or anti-CD86 antibodies could decouple NF-*κ*B activation from caspase-3 cleavage, potentially disrupting the observed inflammatory-apoptotic synergy [62]; (2) Transition phase (3–6 h): Agpat1 agonism [44] may restore mitochondrial cardiolipin homeostasis, suppressing cGAS-STING-mediated delayed apoptosis while preserving energy metabolism [63]; (3) Late phase (6–12 h): Modulation of FZD2 rebalances ceramide/sphingosine-1-phosphate ratios, suppressing NLRP3 [64] inflammasome hyperactivation and reducing MMP-9-mediated blood-brain barrier disruption in progressive ischemic injury [17].

While our integration of characteristic gene mechanisms and experimental validation solidifies the central regulatory role of TLR2/TLR4/NF-*κ*B signaling in hyperacute-phase temporal specificity, emerging crosstalk with parallel pathways—including cGAS-STING, NLRP3 inflammasome, RAGE-HMGB1, and JAK-STAT3—may collectively shape neuroinflammation-apoptosis dynamics. During the early phase (3–6 h), TLR2/TLR4-driven NF-*κ*B activation rapidly induces TNF-*α*/IL-6 release, while TLR4-activated TRIF-TBK1-IRF3 signaling could synergize with subsequent cGAS-STING [65] responses via IFN-*β* priming [66]. Concurrently, the 3 h surge of Cd86 (a TLR4 co-stimulator) potentially amplifies inflammatory cascades through STAT3-TLR4 positive feedback. Transitioning to the mid-phase (6–12 h), Degs2 downregulation may lower NLRP3 activation thresholds via sphingolipid modulation, facilitating TLR-primed inflammasome assembly and IL-1*β* maturation, while Agpat1 suppression at 6 h might link mitochondrial destabilization to delayed cGAS-STING-mediated apoptosis—mechanistically explaining the observed apoptotic fluctuations. Additionally, compensatory networks emerge: NLRP3/caspase-1-mediated MyD88 cleavage could attenuate TLR overactivation [67], and Nlgn2 reduction at 6 h might engage Notch-Hes1 signaling [68] to restrain NF-*κ*B [69]. Although these speculative interactions await experimental confirmation, they outline a spatiotemporal therapeutic blueprint: targeting TLR4/Cd86-MyD88 during 0–3 h to block inflammatory initiation; administering Agpat1 agonists at 3–6 h to stabilize mitochondria and prevent cGAS-STING activation; and employing Degs2 modulators at 6–12 h to balance sphingolipid metabolism and suppress NLRP3 hyperactivation.

In addition, several limitations warrant consideration. First, while our multi-timepoint design captured dynamic molecular changes, clinical comorbidities such as diabetes is known to alter TLR4 expression and apoptotic thresholds [70] were not modeled. Second, medication effects including antiplatelet agents influencing CD86 expression [71] may confound gene expression patterns. Future studies incorporating detailed medication histories and single-cell spatial transcriptomics could delineate how preexisting vascular pathology interacts with hyperacute molecular responses.

**Figure 10:**
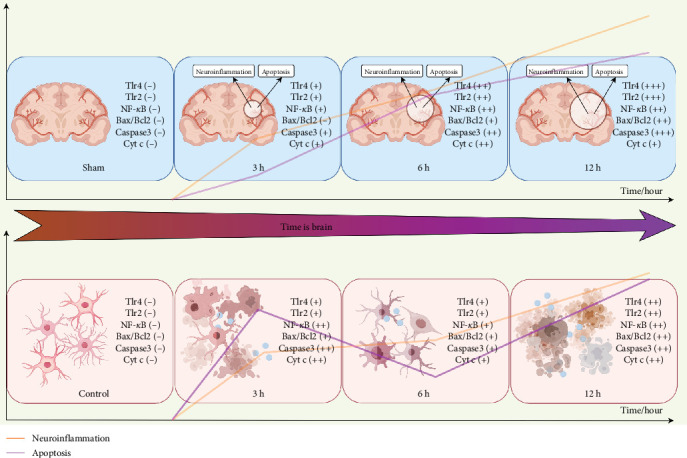
Mechanism diagram.

In summary, our current study systematically identified hyperacute-phase signature genes of AIS through bioinformatic screening, with subsequent validation focusing on the TLR2/TLR4/NF-*κ*B pathway's temporal regulation of apoptosis and neuroinflammation via multi-timepoint in vivo and in vitro assays (Figure 10). Future research should expand to multicenter [72] cohorts integrating advanced imaging biomarkers [73] to validate these signatures across diverse populations, particularly in patients with carotid stenosis-related MMP-9 dynamics [17] and accelerated biological aging phenotypes [25]. While establishing characteristic inflammatory linear progression and revealing a biphasic apoptotic pattern, this work creates a dual-axis mechanistic framework for AIS hyperacute management. The inflammatory axis provides a platform for single-cell spatiotemporal omics to dissect metabolic crosstalk between Pip5k1c-mediated lipid signaling and neutrophil-derived ischemic tolerance factors [9], while the apoptotic axis enables conditional gene editing models targeting Nlgn2-Notch-Hes1 to modulate neurovascular unit responses through synaptic-vascular decoding. Emerging chronotherapeutic strategies, paticularly circadian-regulated NLRP3 inflammasome activity [74], could be synergistically integrated with machine learning-driven biomarker panels [75] to establish spatiotemporal treatment algorithms—optimizing anti-inflammatory interventions during neuroinflammation escalation phases while administering prosurvival therapies at apoptotic troughs. These advanced approaches will ultimately bridge molecular insights (Pip5k1c phospholipid metabolic reprogramming) and clinical implementation (Nlgn2 synaptic plasticity modulator), transforming the observed temporal dissociation between inflammation and apoptosis into chronologically precise intervention windows.

## 5. Conclusion

This study systematically investigates the hyperacute phase of AIS through integrated bioinformatic screening and multi-timepoint experimental validation (3/6/12 h). We identified six signature genes (*Pip5k1c*, *Nlgn2*, *Fzd2*, *Cd86*, *Agpat1*, and *Degs2*) associated with the temporal activation of the TLR2/TLR4/NF-*κ*B pathway, revealing two distinct dynamic patterns: a linear progression of neuroinflammatory responses and a biphasic fluctuation in apoptotic activity. These findings advance our understanding of spatiotemporal heterogeneity between neuroinflammation and apoptosis during the hyperacute therapeutic window, providing foundational evidence for addressing molecular mechanisms underlying time-sensitive interventions in AIS management.

## Figures and Tables

**Table 1:** RT-PCR primers.

Primers	Sequence
Pip5k1c F	5′-ACCTGTTGCCTTCCGTTACTT-3′
Pip5k1c R	5′-GGGGTTCTGGTTGAGGTTCAT-3′
Nlgn2 F	5′-ACTTCGCCAAGACTGGTGAC-3′
Nlgn2 R	5′-ACCTTGTTGGCACGGTAGTT-3′
Fzd2 F	5′-TCTGAGGACGGTTATCGCAC-3′
Fzd2 R	5′-AACCAGGTGAGGGACAGAATC-3′
Cd86 F	5′-AAGGACACGGGCTTGTATGAT-3′
Cd86 R	5′-TAGGTTTCGGGTATCCTTGCT-3′
Agpat1 F	5′-AACGCACAGGAGACGCTATC-3′
Agpat1 R	5′-ATGACAATGGGGACGATGGG-3′
Degs2 F	5′-CTGCCACTGGTACGGATGATT-3′
Degs2 R	5′-CCTCAAACACGAAGTCCCAGA-3′
TLR2 F	5′-GCCCTCAGTCTTGGAGTGTC-3′
TLR2 R	5′-CCTGCTCGCTGTAGGAAACA-3′
TLR4 F	5′-CCAGAGCCGTTGGTGTATCT-3′
TLR4 R	5′-AGAAGATGTGCCTCCCCAGA-3′
NF-*κ*b F	5′-TCACTCGGAGACTGGAACCTG-3′
NF-*κ*b R	5′-GATTTCTTCCCCTCCCGTCA-3′
*β*-actin F	5′-CTCTGTGTGGATTGGTGGCT-3′
*β*-actin R	5′-CGCAGCTCAGTAACAGTCCG-3′

## Data Availability

The data that support the findings of this study are available from the corresponding author upon reasonable request.

## Nomenclature

Agpat1: 1-acylglycerol-3-phosphate O-acyltransferase 1
AIS: acute ischemic stroke
Caspase-3: cysteinyl aspartate specific proteinase 3
CCA: common carotid artery
CCK-8: cell counting kit-8
Cyt-c: cytochrome c
DEGS: differentially expressed genes
Degs2: delta4-desaturase, sphingolipid2
ECA: external carotid artery
ELISA: enzyme-Linked Immunosorbent Assay
FZD2: frizzled class receptor 2
GO: gene ontology
GSEA: gene set enrichment analysis
H&E: hematoxylin and eosin
ICA: internal carotid artery
IF: immunofluorescence
KEGG: Kyoto encyclopedia of genes and genomes
MCA: middle cerebral artery
MMP,△Ψm: mitochondrial membrane potential
MNSS: modified neurological severity score
OGD: oxygen-glucose deprivation
Pip5k1c: phosphatidylinositol-4-phosphate5-kinase Type1 Gamma
PMCAO: permanent middle cerebral artery occlusion
RF: random forest
RFE: recursive feature elimination
ROC: receiver operating characteristic
RT-PCR: real-time quantitative polymerase chain reaction
SVM: support vector machines
TLRs: toll-like receptors
TTC: 2,3,5-tripenyl tetrazolium chloride
TUNEL: terminal deoxynucleotidyl transferase-mediated dUTP Nick end labeling
WB: western blotting
WGCNA: Weighted gene co-expression network analysis

## Supporting Information

Supporting Information 1 Figure S1: Raw and Standardized Data for Gene Expression Validation; Figure S2: Q-PCR Results from In Vivo Experiments; Figure S3: Q-PCR Results from In Vitro Experiments are supporting information provided in response to Point 10 raised by Reviewer 2:"10. For the gene expression validation, include both raw and normalized data in the supporting information for transparency and reproducibility.“ Supporting Information Figure S1 contains the analysis of Pip5k1c, Nlgn2, Fzd2, Cd86, Agpat1, and Degs2 expression in normal and disease groups, derived from the GEO datasets GSE199066, GSE260488, GSE279377, GSE268634, and GSE166162. Supporting Information Figures S2 and S3 provide the raw qPCR data from in vivo and in vitro experiments.

Supporting Information 2 Figure S4: Raw Grey Values from the Western Blot Results is supporting information provided in response to Reviewer 1′s request: “Rigour of statistical analysis: Raw grey values were not provided for the data from the Western blot results (Figures 6, 7, 8) and are recommended to be added in the supporting data.” This file contains the raw grey values from both in vivo and in vitro Western blot experiments.

## References

[1] GBD 2019 Stroke Collaborators (2021). Global, Regional, and National Burden of Stroke and Its Risk Factors, 1990-2019: A Systematic Analysis for the Global Burden of Disease Study 2019. *Lancet Neurology*.

[2] Mendelson S. J., Prabhakaran S. (2021). Diagnosis and Management of Transient Ischemic Attack and Acute Ischemic Stroke: A Review. *JAMA*.

[3] Sacco R. L., Kasner S. E., Broderick J. P. (2013). An Updated Definition of Stroke for the 21st Century: A Statement for Healthcare Professionals From the American Heart Association/American Stroke Association. *Stroke*.

[4] Saver J. L. (2006). Time Is Brain—Quantified. *Stroke*.

[5] Fransen P. S., Berkhemer O. A., Lingsma H. F. (2016). Time to Reperfusion and Treatment Effect for Acute Ischemic Stroke: A Randomized Clinical Trial. *JAMA Neurology*.

[6] Allen L. M., Hasso A. N., Handwerker J., Farid H. (2012). Sequence-Specific MR Imaging Findings That Are Useful in Dating Ischemic Stroke. *Radiographics*.

[7] Ding L., Liu Y., Meng X. (2023). Biomarker and Genomic Analyses Reveal Molecular Signatures of Non-Cardioembolic Ischemic Stroke. *Signal Transduction and Targeted Therapy*.

[8] Han B., Zhang Y., Zhang Y. (2018). Novel Insight Into Circular RNA HECTD1 in Astrocyte Activation Via Autophagy by Targeting MIR*14*2-TIPARP: Implications for Cerebral Ischemic Stroke. *Autophagy*.

[9] Liu R., Luo S., Zhang Y. S., Tsang C. K. (2023). Plasma Metabolomic Profiling of Patients With Transient Ischemic Attack Reveals Positive Role of Neutrophils in Ischemic Tolerance. *Ebiomedicine*.

[10] Pulukool S. K., Srimadh B. S., Vijay S. K. (2024). Noninvasive Cardiac-Specific Biomarkers for the Diagnosis and Prevention of Vascular Stenosis in Cardiovascular Disorder. *Frontiers in Pharmacology*.

[11] Yang K., Zeng L., He Q., Wang S., Xu H., Ge J. (2024). Advancements in Research on the Immune-Inflammatory Mechanisms Mediated by NLRP3 Inflammasome in Ischemic Stroke and the Regulatory Role of Natural Plant Products. *Frontiers in Pharmacology*.

[12] Smith M., Reddy U., Robba C., Sharma D., Citerio G. (2019). Acute Ischaemic Stroke: Challenges for the Intensivist. *Intensive Care Medicine*.

[13] Chen J., Li Y., Wang L. (2001). Therapeutic Benefit of Intravenous Administration of Bone Marrow Stromal Cells After Cerebral Ischemia in Rats. *Stroke*.

[14] Ye X., Shen T., Hu J. (2017). Purinergic 2X7 Receptor/NLRP3 Pathway Triggers Neuronal Apoptosis After Ischemic Stroke in the Mouse. *Experimental Neurology*.

[15] Ma D. C., Zhang N. N., Zhang Y. N., Chen H. S. (2021). Salvianolic Acids for Injection Alleviates Cerebral Ischemia/Reperfusion Injury by Switching M1/M2 Phenotypes and Inhibiting NLRP3 Inflammasome/Pyroptosis Axis in Microglia In Vivo and In Vitro. *Journal of Ethnopharmacology*.

[16] Meng X., Xie W., Xu Q. (2018). Neuroprotective Effects of Radix Scrophulariae on Cerebral Ischemia and Reperfusion Injury via MAPK Pathways. *Molecules*.

[17] Kollikowski A. M., Pham M., Marz A. G. (2024). MMP-9 Release Into Collateral Blood Vessels Before Endovascular Thrombectomy to Assess the Risk of Major Intracerebral Haemorrhages and Poor Outcome for Acute Ischaemic Stroke: A Proof-of-Concept Study. *Ebiomedicine*.

[18] Tong W., Zhang Y., Hui H. (2023). Sensitive Magnetic Particle Imaging of Haemoglobin Degradation for the Detection and Monitoring of Intraplaque Haemorrhage in Atherosclerosis. *Ebiomedicine*.

[19] Jung D. Y., Lee H., Jung B. Y. (2005). TLR4, but not TLR2, Signals Autoregulatory Apoptosis of Cultured Microglia: A Critical Role of IFN-Beta as a Decision Maker. *Journal of Immunology*.

[20] Bai X., Wang X., Lin T. (2022). Toll-Like Receptor 2 is Associated With the Immune Response, Apoptosis, and Angiogenesis in the Mammary Glands of Dairy Cows with Clinical Mastitis. *International Journal of Molecular Sciences*.

[21] Xiong Y., Li S., Wang C. (2025). Chinese Stroke Association Guidelines on Reperfusion Therapy for Acute Ischaemic Stroke 2024. *Stroke Vascular Neurology*.

[22] Lee E. J., Kim S. J., Bae J. (2021). Impact of Onset-To-Door Time on Outcomes and Factors Associated With Late Hospital Arrival in Patients With Acute Ischemic Stroke. *Plos One*.

[23] Mercier E., Boutin A., Lauzier F. (2013). Predictive Value of S-100beta Protein for Prognosis in Patients With Moderate and Severe Traumatic Brain Injury: Systematic Review and Meta-Analysis. *BMJ*.

[24] Yang Z., Wang K. K. (2015). Glial Fibrillary Acidic Protein: From Intermediate Filament Assembly and Gliosis to Neurobiomarker. *Trends in Neurosciences*.

[25] Wang M., Yan H., Zhang Y. (2025). Accelerated Biological Aging Increases the Risk of Short- and Long-Term Stroke Prognosis in Patients With Ischemic Stroke or TIA. *Ebiomedicine*.

[26] Xiong Y., Campbell B., Schwamm L. H. (2024). Tenecteplase for Ischemic Stroke at 4.5 to 24 Hours Without Thrombectomy. *New England Journal of Medicine*.

[27] Yang P., Zhang Y., Liu J. (2025). Ischaemic Stroke in 2024: Progress on Multiple Fronts. *Lancet Neurology*.

[28] Xu J., Wang A., Meng X. (2021). Edaravone Dexborneol Versus Edaravone Alone for the Treatment of Acute Ischemic Stroke: A Phase III, Randomized, Double-Blind, Comparative Trial. *Stroke*.

[29] Wenk M. R., Pellegrini L., Klenchin V. A. (2001). PIP Kinase Igamma is the Major PI(4,5)P(2) Synthesizing Enzyme at the Synapse. *Neuron*.

[30] Wright B. D., Loo L., Street S. E. (2014). The Lipid Kinase PIP5K1C Regulates Pain Signaling and Sensitization. *Neuron*.

[31] Qu M., Chen M., Gong W. (2023). Pip5k1c Loss in Chondrocytes Causes Spontaneous Osteoarthritic Lesions in Aged Mice. *Aging and Disease*.

[32] Jonas K., Prinz F., Ferracin M. (2023). MiR-4649-5p Acts as a Tumor-Suppressive MicroRNA in Triple Negative Breast Cancer by Direct Interaction With PIP5K1C, Thereby Potentiating Growth-Inhibitory Effects of the AKT Inhibitor Capivasertib. *Breast Cancer Research*.

[33] Hamanaka K., Miyake N., Mizuguchi T. (2022). Large-Scale Discovery of Novel Neurodevelopmental Disorder-Related Genes Through a Unified Analysis of Single-Nucleotide and Copy Number Variants. *Genome Medicine*.

[34] Kathuria A., Lopez-Lengowski K., Watmuff B., McPhie D., Cohen B. M., Karmacharya R. (2019). Synaptic Deficits in iPSC-Derived Cortical Interneurons in Schizophrenia are Mediated by NLGN2 and Rescued by N-Acetylcysteine. *Translational Psychiatry*.

[35] Huang L., Luo E. L., Xie J. (2019). FZD2 Regulates Cell Proliferation and Invasion in Tongue Squamous Cell Carcinoma. *International Journal of Biological Sciences*.

[36] Hyun S. Y., Min H. Y., Lee H. J. (2022). Ninjurin1 Drives Lung Tumor Formation and Progression By Potentiating Wnt/Beta-Catenin Signaling Through Frizzled2-LRP6 Assembly. *Journal of Experimental and Clinical Cancer Research*.

[37] Zhou B., Zuo X. X., Li Y. S. (2017). Integration of microRNA and mRNA Expression Profiles in the Skin of Systemic Sclerosis Patients. *Scientific Reports*.

[38] Zhou Y., Li Y., Zhou B. (2017). Inflammation and Apoptosis: Dual Mediator Role for Toll-Like Receptor 4 in the Development of Necrotizing Enterocolitis. *Inflammatory Bowel Diseases*.

[39] Lu H., Ouyang W., Huang C. (2006). Inflammation, a Key Event in Cancer Development. *Molecular Cancer Research*.

[40] Teutsch S. M., Booth D. R., Bennetts B. H., Heard R. N., Stewart G. J. (2004). Association of Common T Cell Activation Gene Polymorphisms With Multiple Sclerosis in Australian Patients. *Journal of Neuroimmunology*.

[41] Matsushita M., Tsuchiya N., Oka T., Yamane A., Tokunaga K. (2000). New Polymorphisms of Human CD80 and CD86: Lack of Association With Rheumatoid Arthritis and Systemic Lupus Erythematosus. *Genes and Immunity*.

[42] Liao W. L., Chen R. H., Lin H. J. (2011). The Association Between Polymorphisms of B7 Molecules (CD80 and CD86) and Graves’ Ophthalmopathy in a Taiwanese Population. *Ophthalmology*.

[43] Ma X. Y., Duan A. Q., Lu X. R. (2022). Novel Insight into the Potential Role of Acylglycerophosphate Acyltransferases Family Members on Triacylglycerols Synthesis in Buffalo. *International Journal of Molecular Sciences*.

[44] Agarwal A. K., Tunison K., Dalal J. S. (2017). Metabolic, Reproductive, and Neurologic Abnormalities in Agpat1-Null Mice. *Endocrinology*.

[45] Mizutani Y., Kihara A., Igarashi Y. (2004). Identification of the Human Sphingolipid C4-Hydroxylase, hDES2, and Its Up-Regulation During Keratinocyte Differentiation. *FEBS Letters*.

[46] Ohi K., Ursini G., Li M. (2015). DEGS2 Polymorphism Associated With Cognition in Schizophrenia is Associated With Gene Expression in Brain. *Translational Psychiatry*.

[47] Hashimoto R., Ikeda M., Ohi K. (2013). Genome-Wide Association Study of Cognitive Decline in Schizophrenia. *American Journal of Psychiatry*.

[48] Kanzler H., Barrat F. J., Hessel E. M., Coffman R. L. (2007). Therapeutic Targeting of Innate Immunity With Toll-Like Receptor Agonists and Antagonists. *Nature Medicine*.

[49] Zhou M., Zhang T., Zhang B. (2022). A DNA Nanostructure-Based Neuroprotectant Against Neuronal Apoptosis Via Inhibiting Toll-Like Receptor 2 Signaling Pathway in Acute Ischemic Stroke. *ACS Nano*.

[50] Caso J. R., Pradillo J. M., Hurtado O., Lorenzo P., Moro M. A., Lizasoain I. (2007). Toll-Like Receptor 4 is Involved in Brain Damage and Inflammation After Experimental Stroke. *Circulation*.

[51] Bell M. T., Puskas F., Agoston V. A. (2013). Toll-Like Receptor 4-Dependent Microglial Activation Mediates Spinal Cord Ischemia-Reperfusion Injury. *Circulation*.

[52] Brodsky I., Medzhitov R. (2007). Two Modes of Ligand Recognition by TLRs. *Cell*.

[53] Durán-Laforet V., Peña-Martínez C., García-Culebras A., Alzamora L., Moro M. A., Lizasoain I. (2021). Pathophysiological and Pharmacological Relevance of TLR4 in Peripheral Immune Cells After Stroke. *Pharmacology and Therapeutics*.

[54] Khandoga A. G., Khandoga A., Anders H. J., Krombach F. (2009). Postischemic Vascular Permeability Requires Both TLR-2 and TLR-4, but Only TLR-2 Mediates the Transendothelial Migration of Leukocytes. *Shock*.

[55] Sun P. P., Yuan F., Xu J., Sai K., Chen J., Guan S. (2014). Cryptotanshinone Ameliorates Hepatic Normothermic Ischemia and Reperfusion Injury in Rats by Anti-Mitochondrial Apoptosis. *Biological and Pharmaceutical Bulletin*.

[56] Lee E. F., Fairlie W. D. (2019). The Structural Biology of Bcl-x(L). *International Journal of Molecular Sciences*.

[57] Ola M. S., Nawaz M., Ahsan H. (2011). Role of Bcl-2 Family Proteins and Caspases in the Regulation of Apoptosis. *Molecular and Cellular Biochemistry*.

[58] Cunningham P. N., Wang Y., Guo R., He G., Quigg R. J. (2004). Role of Toll-Like Receptor 4 in Endotoxin-Induced Acute Renal Failure. *Journal of Immunology*.

[59] Wang P., Qiu W., Dudgeon C. (2009). PUMA is Directly Activated by NF-KappaB and Contributes to TNF-Alpha-Induced Apoptosis. *Cell Death and Differentiation*.

[60] Erickson E. K., Blednov Y. A., Harris R. A., Mayfield R. D. (2019). Glial Gene Networks Associated With Alcohol Dependence. *Scientific Reports*.

[61] Barnett K. C., Coronas-Serna J. M., Zhou W. (2019). Phosphoinositide Interactions Position cGAS at the Plasma Membrane to Ensure Efficient Distinction Between Self- and Viral DNA. *Cell*.

[62] Gong P., Jia H. Y., Li R. (2023). Downregulation of Nogo-B Ameliorates Cerebral Ischemia/Reperfusion Injury in Mice Through Regulating Microglia Polarization Via TLR4/NF-kKappaB pPathway. *Neurochem International*.

[63] Du Q., Ning N., Zhao X. (2025). Acylglycerol Kinase Inhibits Macrophage Anti-Tumor Activity Via Limiting mtDNA Release and cGAS-STING-Type I IFN Response. *Theranostics*.

[64] Villasenor T., Madrid-Paulino E., Maldonado-Bravo R., Urban-Aragon A., Perez-Martinez L., Pedraza-Alva G. (2017). Activation of the Wnt Pathway by Mycobacterium Tuberculosis: A Wnt-Wnt Situation. *Frontiers in Immunology*.

[65] Pan W. Q., He Y. H., Su Q. (2016). Association of Decreased Serum Vasostatin-2 Level With Ischemic Chronic Heart Failure and With MACE in 3-Year Follow-up: Vasostatin-2 Prevents Heart Failure in Myocardial Infarction Rats. *International Journal of Cardiology*.

[66] Tesser A., Piperno G. M., Pin A. (2021). Priming of the cGAS-STING-TBK1 Pathway Enhances LPS-Induced Release of Type I Interferons. *Cells*.

[67] Larabi A., Barnich N., Nguyen H. (2020). New Insights Into the Interplay Between Autophagy, Gut Microbiota and Inflammatory Responses in IBD. *Autophagy*.

[68] Saint-Martin M., Goda Y. (2023). Astrocyte-Synapse Interactions and Cell Adhesion Molecules. *The FEBS Journal*.

[69] Maniati E., Bossard M., Cook N. (2011). Crosstalk Between the Canonical NF-KappaB and Notch Signaling Pathways Inhibits Ppargamma Expression and Promotes Pancreatic Cancer Progression in Mice. *Journal of Clinical Investigation*.

[70] Wada J., Makino H. (2016). Innate Immunity in Diabetes and Diabetic Nephropathy. *Nature Reviews Nephrology*.

[71] Simoni O., Scarpa M., Castagliuolo I. (2024). IMMUNOREACT 7: Regular Aspirin Use is Associated With Immune Surveillance Activation in Colorectal Cancer. *Cancer*.

[72] van Dam-Nolen D., Truijman M., van der Kolk A. G. (2022). Carotid Plaque Characteristics Predict Recurrent Ischemic Stroke and TIA: The PARISK (Plaque At RISK) Study. *Jacc Cardiovascular Imaging*.

[73] Irwin D. J., Fedler J., Coffey C. S. (2020). Evolution of Alzheimer’s Disease Cerebrospinal Fluid Biomarkers in Early Parkinson’s Disease. *Annals of Neurology*.

[74] Eckle T., Bertazzo J., Khatua T. N. (2024). Circadian Influences on Myocardial Ischemia-Reperfusion Injury and Heart Failure. *Circulation Research*.

[75] Qin X., Ding R., Lu H. (2024). Identification of Pivotal Genes and Regulatory Networks Associated With Atherosclerotic Carotid Artery Stenosis Based on Comprehensive Bioinformatics Analysis and Machine Learning. *Frontiers in Pharmacology*.

